# VIOLA—A Multi-Purpose and Web-Based Visualization Tool for Neuronal-Network Simulation Output

**DOI:** 10.3389/fninf.2018.00075

**Published:** 2018-11-08

**Authors:** Johanna Senk, Corto Carde, Espen Hagen, Torsten W. Kuhlen, Markus Diesmann, Benjamin Weyers

**Affiliations:** ^1^Institute of Neuroscience and Medicine (INM-6), Institute for Advanced Simulation (IAS-6), JARA Institute Brain Structure-Function Relationships (INM-10), Jülich Research Centre, Jülich, Germany; ^2^Visual Computing Institute, RWTH Aachen University, Aachen, Germany; ^3^JARA - High-Performance Computing, Aachen, Germany; ^4^IMT Atlantique Bretagne-Pays de la Loire, Brest, France; ^5^Department of Physics, University of Oslo, Oslo, Norway; ^6^Department of Psychiatry, Psychotherapy and Psychosomatics, Medical Faculty, RWTH Aachen University, Aachen, Germany; ^7^Department of Physics, Faculty 1, RWTH Aachen University, Aachen, Germany

**Keywords:** interactive visualization, visual data analytics, coordinated multiple views, 3D visualization, neuronal network simulation, spiking neurons, spatiotemporal patterns, data analysis workflow

## Abstract

Neuronal network models and corresponding computer simulations are invaluable tools to aid the interpretation of the relationship between neuron properties, connectivity, and measured activity in cortical tissue. Spatiotemporal patterns of activity propagating across the cortical surface as observed experimentally can for example be described by neuronal network models with layered geometry and distance-dependent connectivity. In order to cover the surface area captured by today's experimental techniques and to achieve sufficient self-consistency, such models contain millions of nerve cells. The interpretation of the resulting stream of multi-modal and multi-dimensional simulation data calls for integrating interactive visualization steps into existing simulation-analysis workflows. Here, we present a set of interactive visualization concepts called views for the visual analysis of activity data in topological network models, and a corresponding reference implementation VIOLA (VIsualization Of Layer Activity). The software is a lightweight, open-source, web-based, and platform-independent application combining and adapting modern interactive visualization paradigms, such as coordinated multiple views, for massively parallel neurophysiological data. For a use-case demonstration we consider spiking activity data of a two-population, layered point-neuron network model incorporating distance-dependent connectivity subject to a spatially confined excitation originating from an external population. With the multiple coordinated views, an explorative and qualitative assessment of the spatiotemporal features of neuronal activity can be performed upfront of a detailed quantitative data analysis of specific aspects of the data. Interactive multi-view analysis therefore assists existing data analysis workflows. Furthermore, ongoing efforts including the European Human Brain Project aim at providing online user portals for integrated model development, simulation, analysis, and provenance tracking, wherein interactive visual analysis tools are one component. Browser-compatible, web-technology based solutions are therefore required. Within this scope, with VIOLA we provide a first prototype.

## 1. Introduction

One common technique to capture brain activity on the neuronal level is to record extracellular potentials in cortical tissue (Buzsáki et al., 2012; Einevoll et al., 2013). The low frequency (≲100 Hz) part of the signal, often referred to as the local field potential (LFP), remains difficult to interpret as thousands to millions of proximal and distal neurons contribute to the signal (Kajikawa and Schroeder, 2011; Lindén et al., 2011; Łeski et al., 2013). From the high-frequency band (≳100 Hz), however, one can detect sequences of spikes, the transient extracellular signatures of action potentials in single neurons nearby the recording electrode. The number of reliably identified neurons (through spike sorting, Quiroga, 2007) per recording session is low compared to the number of neurons in vicinity of the recording device, even if the experiment is performed with hundreds or more electrode contact points (Einevoll et al., 2012). The Utah array from Blackrock Microsystems^1^, for example, resolves with 10 × 10 electrodes on 4 × 4 mm^2^ little more than a hundred distinct neurons. Also optical methods for measuring neuronal activity have seen continuous improvements. As recently demonstrated, non-invasive three-photon fluorescence microscopy facilitates functional imaging at high optical resolution as deep as 1 mm (Ouzounov et al., 2017). While the method simultaneously images a comparably large number of neurons, the recordings lack the temporal resolution to reliably detect individual action potentials. Ouzounov et al. (2017) record from as many as 150 neurons in mouse hippocampal stratum pyramidale within a field of view of 200 × 200 μm^2^.

The rapidly improving parallel recording technology increases the need for suitable analysis methods for high-dimensional and dynamic data streams. Nevertheless, the recordings will remain to be characterized by a massive undersampling for some time. Therefore, detailed full scale models of the cortical tissue are required to understand the microscopic dynamics (van Albada et al., 2015) and to relate the microscopic activity to mesoscopic measures like the LFP. For this program to succeed, neuroscientists not only need to analyze model data in the same way as experimental data, but to explore data sets with orders of magnitude more channels and more modalities than experimentally available.

Networks of model neurons incorporating varying levels of biophysical and anatomical detail reproduce a number of features of experimentally obtained spike trains. For networks of point- or one-compartment neuron models, this list of features includes irregular spike trains (Softky and Koch, 1993; van Vreeswijk and Sompolinsky, 1996; Amit and Brunel, 1997; Shadlen and Newsome, 1998), asynchronous spiking (Ecker et al., 2010; Renart et al., 2010; Helias et al., 2014; Ostojic, 2014), correlation structure (Okun and Lampl, 2008; Gentet et al., 2010; Helias et al., 2013), self-sustained activity (Ohbayashi et al., 2003; Kriener et al., 2014), realistic firing rates across cortical lamina (Potjans and Diesmann, 2014), single-neuron spiking activity of different cell types (Izhikevich, 2003; Kobayashi et al., 2009; Yamauchi et al., 2011) and responses under “*in vivo*” conditions (Jolivet et al., 2008; Gerstner and Naud, 2009). Relating point-neuron network activity to population signals such as the LFP is, however, not straightforward. Approximations (see Mazzoni et al., 2015) or forward-model based schemes (Hagen et al., 2016) are required to bridge the gap to experimental electrophysiological data which predominantly reflects population activity.

The focus of this study lies on visualization methods for activity of spatially extended neuronal network models. Incorporation of spatial structure is a prerequisite for models aiming to explain experimentally observed spatiotemporal patterns of activity (Rubino et al., 2006; Denker et al., 2011; Sato et al., 2012; Muller et al., 2014; Townsend et al., 2015). Such models have an arrangement of neurons in one-, two-, or three-dimensional (1D, 2D, or 3D) space and connection rules which typically depend on the distance between (parts of) the neurons (Mehring et al., 2003; Coombes, 2005; Yger et al., 2011; Bressloff, 2012; Voges and Perrinet, 2012; Kriener et al., 2014; Keane and Gong, 2015; Rosenbaum et al., 2017). Although we primarily focus on model data, the same visualization methods can be applied with experimentally recorded data.

Decades of research in computational neuroscience have left the field without widely used visualization platforms and standards (Lansner and Diesmann, 2012). This is unlike the situation for simulation technology in computational neuroscience. Here, mature and sustainable codes have emerged and are in continuous development and use by large communities (Carnevale and Hines, 2006; Gewaltig and Diesmann, 2007; Goodman and Brette, 2009). The origins of this discrepancy may have technological, sociological, as well as funding aspects and an in-depth analysis is beyond the scope of the present manuscript. However, the architectural changes over the years and the progress in visualization concepts reported here may already shed some light on the deeper question.

As a reference implementation of this conceptual study, we introduce the interactive visualization tool VIOLA, an open-source, platform-independent, and lightweight web-browser application. The tool is designed for initial visual inspection of massively parallel data generated primarily by simulations of spatially organized spiking neuronal networks. VIOLA is designed around general principles of information visualization (Shneiderman, 1996; Wang Baldonado et al., 2000) and its 2D and 3D visualizations support the exploration of neuronal activity across space and time. The software can display raw spiking output as well as spatiotemporally binned data that may represent instantaneous spike counts gathered from nearby groups of neurons. Spike and LFP data can be displayed simultaneously, thus allowing for a multi-modal analysis.

The manuscript is organized as follows: In Section 2 we present different visualization types and their application. In Section 3 we subsequently describe their implementation in the visualization tool VIOLA, an example network model, and the phenomenological model for the LFP signal. Finally, in Section 4, we conclude our work and discuss general limitations of frameworks for explorative visualization and potential future developments. Readers primarily interested in the use of each visualization type may hereon choose to skip directly to Section 2. Next, we review common visualization methods for activity in neural networks and the history of visualization in computational neuroscience to provide the background for our considerations.

### 1.1. State-of-the-Art and Historical Perspective

**Figure 1:**
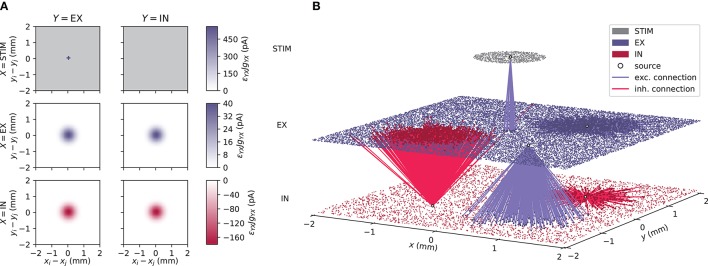
Geometry and connectivity of a layered point-neuron network. **(A)** Schematic illustration of distance-dependent network connectivity using connectivity pattern tables (Nordlie and Plesser, 2010). Each row represents source populations *X* ∈ {STIM, EX, IN}, and each column target populations *Y* ∈ {EX, IN}. The color coding in each image shows the connection intensity between presynaptic neurons *j* and postsynaptic neurons *i* located in (*x*_*j*_, *y*_*j*_) and (*x*_*i*_, *y*_*i*_) with origin (0, 0) at the center. The connection intensities are defined as the product between pairwise connection probabilities ϵ_*YX*_(*r*_*ij*_) and synapse strengths *g*_*YX*_*J* for each respective connection. Gray values denote connection intensities of zero. **(B)** Illustration of one network instantiation with randomly drawn neuron positions and outgoing connections from a subset of neuronal units. The colored dots represent individual units at their (*x, y*)-coordinates. Gray dots denote units in a stimulus (STIM) layer, blue dots excitatory (EX) units, and red dots inhibitory (IN) units. Blue and red lines denote excitatory and inhibitory connections respectively, from a source unit (white circles) onto neurons within the same or another layer.

We here consider typical visualization of activity in an example spiking point-neuron network consisting of an excitatory (EX), an inhibitory (IN), and an external stimulus (STIM) population. EX and IN units are positioned randomly within square domains while STIM units are randomly positioned within a circle at the center. A schematic representation of the network connectivity is shown in Figure 1A. We use connectivity pattern tables (Nordlie and Plesser, 2010) for source populations *X* (rows) and target populations *Y* (columns). The images indicate the “connection intensities” for each connection, defined as the product between averaged pairwise connection probabilities ϵ_*YX*_(*r*_*ij*_) and synapse strengths *g*_*YX*_*J*. The distance between a source and a target neuron is denoted by *r*_*ij*_. Pairwise connection probabilities decay with horizontal distance between EX and IN units according to a Gaussian profile, while STIM units only connect locally to the EX population restricted by a cut-off radius. The geometry of one network instantiation is depicted in Figure 1B. EX (blue dots), IN (red dots), and STIM (gray dots) units are placed in separate layers. The distance dependency is illustrated by outgoing excitatory connections (blue lines) from single units in the STIM and EX populations and outgoing inhibitory connections (red lines) from single units in the IN population.

**Figure 2:**
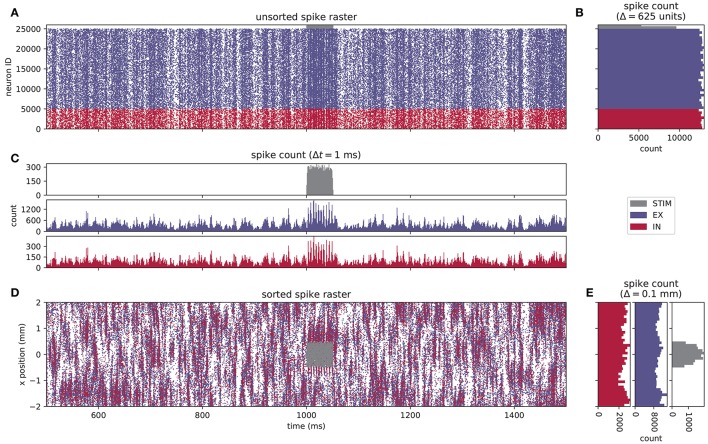
Spiking activity of a layered point-neuron network model. **(A)** Spike raster plot for STIM (gray dots), EX (blue dots), and IN (red dots) units from a simulation of the network instantiation depicted in Figure 1B. Each individual dot corresponds to a unit ID vs. spike time; only the spikes of every fifth neuron are shown in the raster. The color coding for each population is reused in the subsequent panels. **(B)** Spike count histogram across units in each population, calculated using a bin width of 625 units, sorted by neuron index *j*. **(C)** Spike count histogram for each population across time, computed using a temporal bin width Δ*t* = 1 ms. **(D)** Sorted spike raster where dots correspond to the spatial location (projected onto the *x*−axis) and spike times of each unit. The raster-plot density is diluted as in **(A)**. **(E)** Spike count histogram across spatial bins with a width Δ*l* = 0.1 mm.

The visualization of neuronal activity data poses challenges due to the high dimensionality and time dependence of the data. Historically, electrophysiological data have been recorded from few electrodes or from many electrodes with undefined relative and absolute spatial coordinates (see, for example, the pioneering work of Krüger and Bach, 1981). This is not a limitation for recordings within the local cortical network where a neuron can form a synapse with any other neuron and there is little spatial organization. Furthermore, the fundamental interaction in a neuronal network is considered to be a dynamics on a graph; nodes solely interact via the edges of the graph. In this picture the spatial embedding of the graph is arbitrary as the dynamics are not constrained by the dimensions of physical space. Therefore, neuroscientists developed concepts for visualizing correlation structure and time dependence of neuronal activity in multi-channel recordings in ignorance of spatial properties. A temporal segment of activity of our example network is visualized in Figure 2. Figure 2A is the spike raster diagram or dot display in use for decades (explained in Abeles, 1982). Each row shows the spike train of one neuron where spike times are marked by dots. The rows either represent data of the same neuron in several trials or, as here, data of simultaneously recorded neurons in a single trial (Grün, 1996, Figure 6.2). The spike trains are vertically arranged by neuron ID and in addition color coded by population. The spike raster highlights global features of network activity and generations of neuroscientists have been trained to visually inspect these diagrams. For example, network synchrony appears as a stripy vertical pattern even if individual neurons only rarely participate in a synchronous event. The activation of the stimulus population is reflected in the other populations as an increased density of the dots. Epping et al. (1984) extend the concept of the raster diagram by assigning a unique color to the dots of a neuron. In this way multi-channel activity observed over multiple trials can be superimposed. Figure 2B shows spike counts along the temporal axis over neuronal units demonstrating that the per-neuron spike count is similar for the excitatory and the inhibitory populations. The spike count along the vertical axis in Figure 2C is called the post-stimulus time histogram (PSTH, Perkel et al., 1967a), originally computed for an individual neuron observed over several trials. Later the display was also called peri-stimulus time histogram. Here the histogram is computed over simultaneously recorded neurons in a single trial. The display uncovers the fluctuations of population activity in time.

The development of adequate visualization concepts for multi-channel neuronal data is an ongoing endeavor (Allen et al., 2012). The cross-correlation function (Perkel et al., 1967b) exposes the time-averaged relationship between the spike times of two neurons. The snowflake diagram generalizes the concept to three neurons (Perkel et al., 1975; Czanner et al., 2005). Gravitational clustering (Gerstein and Aertsen, 1985; Gerstein et al., 1985; reviewed in Chapter 8 of Grün and Rotter, 2010) attempts to identify the emergence of correlated groups of neurons, so called cell-assemblies, and the temporal dynamics of the changing membership of individual neurons in such groups without averaging over trials. The joint peri-stimulus time histogram (JPSTH, Aertsen et al., 1989) generalizes the cross-correlation function to visualize the dynamics of the correlation between the spikes of two neurons in reference to a stimulus. Later Prut et al. (1998) used the idea to investigate the occurrence of spatiotemporal patterns in the spike trains of three neurons, where “spatio” refers to the abstract space of neuron IDs instead of physical space. Because of the difficulties in determining statistical significance, Grün et al. (2002) restricted the scope to patterns in the space of IDs and for visualization mapped significant events, so called unitary events, back into the spike raster diagram. Progress in the theory of neuronal networks showed that propagating spiking activity due to the stochastic nature of neuronal activity is likely to exhibit in each instance a random sub-pattern of spikes of some superset of neurons. Therefore, Schrader et al. (2008) designed a matrix spanned by binned ongoing time in both dimensions where matrix-elements represent the cardinality of the intersection set of the neurons spiking at the two respective time bins. With color-coded cardinality, in this matrix repeatedly occurring propagating spiking activity appears as a diagonal stripe on a noisy background. Torre et al. (2016a) recently added assessment of statistical significance to the method. Kemere et al. (2008) employ multi-channel recordings to construct the time course of a multi-dimensional vector of spike rates. A suitable projection to a lower dimensional space reveals differentiable trajectories of network activity depending on the experimental protocol (reviewed in Cunningham and Byron, 2014). Another line of work attempts to cope with the combinatorial explosion of patterns in multi-channel spike trains while maintaining sensitivity by the construction of a pattern spectrum: a two-dimensional histogram spanned by the number of spikes in a pattern, called pattern complexity, and the number of occurrences of the particular patterns (Gerstein et al., 2012; Torre et al., 2013).

Today, electrodes can be inserted into the brain in spatially well-defined arrangements and cover distances at which the spatial organization of the tissue is exhibited as in our example in Figure 2. Thus, neurons are identified not only by a unique neuron ID anymore but additionally by their position in physical space. Since the raster diagram in Figure 2A does not provide the researcher with all available information about the recording situation, Figure 2D modifies the diagram to arrange the spike trains on the vertical axis according to the *x*-coordinate of the position of the emitting neuron. In contrast to the regular spike raster, we observe inhomogeneous spatiotemporal features in network activity. The spatially binned spike counts along the temporal axis in Figure 2E, however, do not reveal any unexpected structure. Thus, taking into account one coordinate of the neurons in physical space hints at some organization of neuronal activity. Nevertheless, a higher-dimensional analysis seems to be required to uncover its origin, as the features of spatiotemporal patterns in 2D can only be conjectured.

The emergence of planar wavelike spiking activity in 2D networks was shown by Voges and Perrinet (2012, Figures 3–5), but a 2D spatial visualization of the data could not faithfully capture intermediate mixed patterns such as rings and spiraling waves. Temporal snapshots of spatial activity show the evolution of patterns, as seen in Mehring et al. (2003, Figure 5), Yger et al. (2011, Figures 2, 12), Voges and Perrinet (2012, Figure 6), and Keane and Gong (2015, Figure 1). Series of such snapshots combined in an animation or movie can be informative, but require settings to be defined beforehand, leaving only little room for interactivity. With such non-interactive visualization methods, crucial decisions about a figure or an animation thus have to be made before a sufficient intuition about the data exists. Flexible, interactive visualization techniques identifying relevant dynamical features present in the data would have the potential to avoid the tedious and time-consuming loop of refining parameters and regenerating snapshots, animations, or movies. In addition, high-dimensional and multi-modal data demand adequate workflows for analysis, from raw data to statistical measures, where interactive visual analysis methods can play a major role. It is for example essential to get a basic understanding of the datasets to better decide what statistical methods to use for more elaborate analysis. Furthermore, interactive visualization allows for explorative data analysis, including dimensionality reduction of complex datasets, highlighting of data points, and direct changes to visualization parameters.

For the development of supportive visual analytics tools, Shneiderman (1996) introduced the so-called “information-seeking mantra.” It describes the steps of common visual analysis workflows: “overview first, zoom and filter, details on demand.” The first step provides a superficial “overview”of the data. In the second step, “zooming” into the dataset allows the user to get a more detailed view on a chosen data subset. Application of “filters” implies a change in dimensionality of the data or the extraction of particular features. Finally, Shneiderman (1996) proposes that visualization tools should enable the user to access all details of selected data points.

To not restrict the user to only one visual representation of the data, Wang Baldonado et al. (2000) established the concept of “coordinated multiple views.” Coordinated multiple views is a paradigm for the implementation of visual analysis applications that “use two or more distinct views to support the investigation of a single conceptual entity” (Wang Baldonado et al., 2000, page 110). The paradigm has been applied in various contexts (see for example Roberts, 2007). Basic coordination of views addresses selection operations (e.g., whether to display only a subset of the data) and also includes immediate control over animated frames (e.g., animation time step and playback speed for time-resolved data). In addition, each view may have an exclusive (view-specific) set of user controls and settings.

The activity exhibited by our example network is characterized by a non-trivial interplay between neuronal populations resulting in non-stationary activity in time and space. Let's assume: The neuroscientist needs to identify the propagation of spiking activity within and across individual layers over time and space, and simultaneously observe population activity measures such as the LFP. This is an opportunity to exercise the concepts by Shneiderman (1996) and Wang Baldonado et al. (2000). Visualization in most cases focuses on a specific aspect or hypothesis to be tested by analyzing the corresponding data. Therefore, for each task the neuroscientist combines a different set of views. Particular views are frequently created *ad hoc* specifically for the research question or experimental protocol, as they may not be provided by the visualization framework in use. Hence, the analysis software environment needs to facilitate fast prototyping of visualizations and an interface to a computing programming language used in the scientific domain. This focus on a specific aspect under investigation by the neuroscientist necessarily entails an individual level of reduction or aggregation of the data. A particular visualization realizes this preprocessing of the data with methods like binning of data points in time or space, or by filtering out a certain subset of parameters of each data point. For instance, the spike raster plot in Figure 2A displays the individual spikes of all neurons whereas the bar chart in Figure 2C visualizes the total number of spikes per time step. The visualization abstracts away from the spikes of individual neurons and turns the focus to the whole population. On the one hand the visualization simplifies interpretation by presenting less detail, on the other hand the reduction increases the chance of wrong or inaccurate conclusions. Historically, Vaadia et al. (1988, Figure 4 middle) illustrate a potential misinterpretation of the PSTH due to variability in the onset of the neuronal response: a neuron observed over multiple trials exhibits in the PSTH a smooth increase in spike rate, whereas the raster plots show in each trial an abrupt increase in spike density with a variable onset. Grün et al. (2002, Figure 8) demonstrate how such misalignments can propagate to measures of statistical significance: with respect to one trigger event the data show surplus spike synchrony simply due to non-stationarity of spike rate, whereas with respect to another trigger the rate is stationary and no excess synchrony is detected. However, if a multi-view approach is implemented that combines various visualizations, more than one aspect of the data (more than one visual representation of differently processed data) can be inspected simultaneously and can be put into relation. By interactive addition and removal of certain views, this process can be made flexible and thus address changes in the analysis goals or to consider findings during the analysis. Finally, we require a solution that allows for integration with platform-independent web-based technologies to keep the accessibility of the tool as high as possible.

A variety of coordinated multi-view applications for the interactive analysis of activity data has been described in literature, which generally follow the information-seeking mantra. For models of neuronal systems, the NEURON simulation environment (Carnevale and Hines, 2006) provides a graphical user interface based on a modified version of the discontinued InterViews library in addition to scripting in HOC and Python (Hines et al., 2009). The software itself offers the possibility of drawing multiple concurrent windows with dynamic and interactive plots of voltages, currents, morphology shapes, and phase planes that are updated while simulations of single-neuron models or neuron networks are running. 3D visualization is not directly supported, but NEURON's Python bindings also allow running simulations to interact with modern visualization software, as for example incorporated by NeuronVisio (Mattioni et al., 2012) that relies on the OpenGL-accelerated Mayavi visualization toolset (Ramachandran and Varoquaux, 2011). MOOSE^2^, another neural simulation environment, has a scripting interface with Python and graphical displays based on Matplotlib, PyQt, and OpenGL. The simulation software for large-scale neuronal network models NEST^3^ (NEural Simulation Tool, Gewaltig and Diesmann, 2007) does not provide built-in interactive visualization. The original authors state this in their first report (Diesmann et al., 1995) as a design decision based on two considerations. First, in 1995 the life time of graphics frameworks and libraries appeared much shorter than the envisioned period of relevance of a simulation code. Thus, only a software stack with a strict separation of levels would ensure platform independence and sustainability of NEST. Second, a basic idea of the project is to contribute to a software environment for “*in virtu*” now often called “*in silico*” experiments (restated in Diesmann and Gewaltig, 2002). In the concept of in virtu experiments, the authors state, simulated data should be analyzed with the same analysis tools as experimental data to maximize comparability and reproducibility. At the same time researchers at the department of Physiology and the Center for Neural Computation of the Hebrew University in Jerusalem started to work on an integrated analysis and visualization platform based on Open Inventor^4^ called Neural Data Analysis (NDA) but the project was abandoned with the advent of MATLAB (Vaadia, 2017). Recently, Nowke et al. took on the challenge to develop a simulator independent visualization platform for brain-scale neuronal networks. The VisNEST (Nowke et al., 2013, 2015) framework visualizes the spiking activity of multi-area network models (using as an example Schmidt et al., 2017) in a virtual environment. The time-resolved activity data is mapped onto a 3D brain model. The mapping enables the researcher to interact with the model in 3D to expose otherwise occluded parts of the brain and to relate brain activity to anatomy. In a different view, a dynamic 3D graph represents the time course of spike exchange between different cortical areas. This representation of spatial information can be combined with classic charts such as spike raster plots. The tool does presently not account for the spatial organization of activity within brain areas. Apart from VisNEST, other standalone interactive multi-view applications have been developed for simulated spiking data, for instance SNN3DViewer (Kasiński et al., 2009) and ViSimpl (Galindo et al., 2016). SNN3DViewer focuses on 3D neuronal networks by visualizing individual neurons and their connections schematically, including interactive control over the 3D visualization (navigation, scale). ViSimpl combines a 3D particle-system-based visualization of the simulated neuronal network using color coding for the activity, supplemented by a set of data charts for single neurons and populations. Geppetto^5^ is a web-based modular platform for visualization and simulation of complex biological systems including spiking neuronal networks. Unlike the visualization concepts along which these tools have been developed, we here focus on concepts that expose the spatial organization of neuronal activity in layered networks and scale to signals from several square millimeters of brain surface.

Beside the aforementioned softwares applicable with spike data, general-purpose multi-view frameworks exist with different design goals and contexts of use (Roberts, 2007). One generic high-level example is GLUE^6^, a Python and OpenGL-based multi-view framework. Another powerful framework is the now neglected OpenDX^7^.

The present study aims at web-based visualization. Easy access to libraries of common plotting functions and methods (scatter, line, surface plots etc.) is provided for most common programming languages (C++, Python, MATLAB, etc.). Nevertheless, a large amount of time and resources is still required to construct fully interactive visualization tools adhering to the principles outlined by Shneiderman (1996) and Wang Baldonado et al. (2000). Including interactivity and time synchronization between different visualizations may be demanding in terms of software design and development time, however, existing plotting libraries can be used to realize the individual visualizations.

## 2. Results

For the analysis of data, static figures can help to highlight certain characteristics of the data or show results relevant for a particular hypothesis. However, static figures hamper an exploratory analysis of data as the adaption of data filters, visualization parameters, or changes in the perspective (in case of 3D visualization) require a re-rendering of the figure resulting in a very slow visual analysis process. Interactive visualization tools tackle these shortcomings by offering multiple views on the same data simultaneously, for example by projecting the data across different dimensions. This allows the user to investigate data at different levels of detail, and to adapt visualization parameters in a dynamic and explorative manner as the rendering of the visualization is continuously updated. Throughout this section, we use the spike output of a spatially extended point-neuron network as an example to demonstrate appropriate visualization types in an interactive and multi-view framework (see also Video 1 in the Supplementary Material). The example network is introduced in Section 1.1 with a network illustration in Figure 1 and an example of conventional activity visualization in Figure 2. Neurons in this network are placed in 2D sheets, and connections are drawn using distance-dependent probabilities between pairs of neurons. The model represents spatially heterogeneous neuronal activity across a 4 × 4 mm^2^ cortical sheet. As we here focus on visualization methods in VIOLA, we refer the reader to Sections 3.4 and 3.5 for the details on our network implementation in NEST (Kunkel et al., 2017), Python-based preprocessing steps and predictions of a mesoscopic population signal, the local field potential (LFP). We next describe in detail the different views of VIOLA and their use cases.

### 2.1. Views of VIOLA

**Figure 3:**
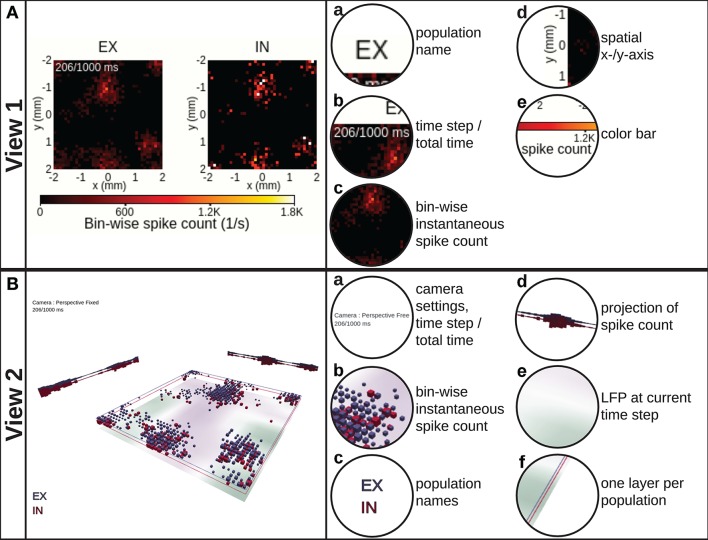
**View 1: 2D spike-count rate. (A)** shows the instantaneous spike-count rates, defined as the number of spikes per second occurring within a spatiotemporal bin, using 2D image plots spanning the spatial *x*− and *y*−axes of the network layers. One separate image plot is created for each network population and denoted by the population name (EX, IN). The color map and corresponding color bar for instantaneous spike-count rate values are shared among all populations. In this and subsequent panels, we show the screen shot of the view itself to the left and highlight its components to the right. **View 2: 3D layered spike-count rate. (B)** combines the data shown in **(A)** in a single 3D scene by stacking the different population data on top of each other. The size of each cubic marker denotes the magnitude of the corresponding bin-wise instantaneous spike count, and its position corresponds to the spatial positions of the respective bin. Unique colors are assigned to each layer as indicated by the population names. Projections of the spike counts along the *x*− and *y*−axes are displayed toward the corresponding edges. The optional bottom image plot layer shows the spatial variation in an LFP-like signal at the present time step of the rendering loop.

**Figure 4:**
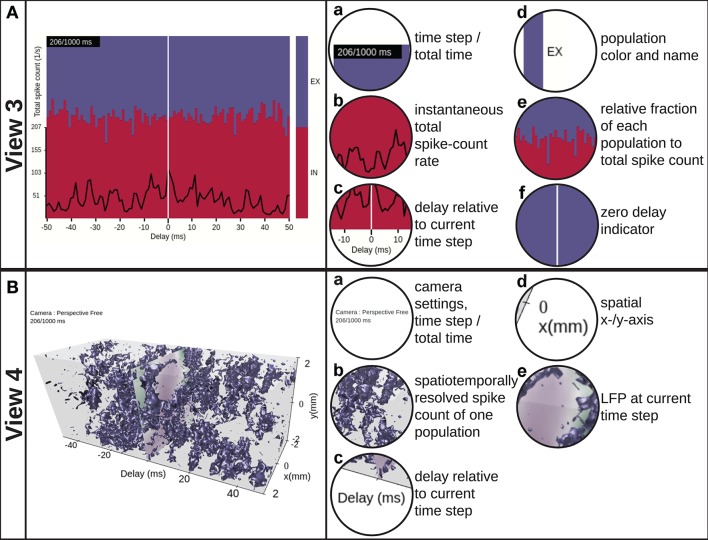
**View 3: Scrolling spike-count rate plot. (A)** is a time-series representation of the data across a user-variable time interval around the present time step of VIOLA's rendering loop, indicated by the vertical white line. The instantaneous total spike-count rate summed over all populations is drawn using a black line. The relative fraction of spikes of each population to the total spikecount rate is shown as a stacked, normalized histogram. The population outputs are color coded as in view 2. **View 4: Scrolling spike-count rate iso-surface plot. (B)** provides a 3D representation of the spatiotemporally resolved spike-count rates of one selected network population across a user-variable time window. The spike-count rate is rendered as a closed iso-value surface in the color of the respective population and extends in both space (*x*− and *y*−axes) and time (delay axis). The present time step in the visualization is indicated by a time lag of zero on the time-delay axis. At zero time delay we also show the LFP signal corresponding to the present time step in the animation.

**Figure 5:**
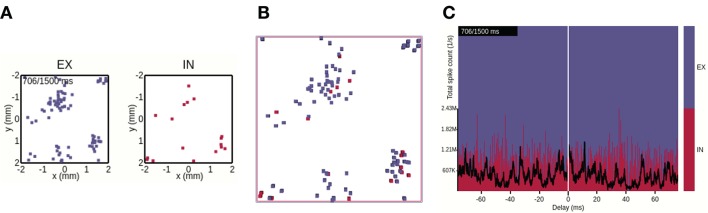
Views 1–3 applied to raw data formats. **(A)** View 1: Each dot corresponds to a single spike event of a neuronal unit at its spatial location in the network. **(B)** View 2: Perspective top-down view onto stacked layers of **(A)**. **(C)** View 3: The stacked plot has the temporal bin-size of the simulation resolution. The black trace shows the spike count of all neurons per time bin (in units of spikes/s).

VIOLA incorporates two conceptually different visualization types with two separate “views” each. The first visualization type (view 1 and view 2 in Figure 3) focuses on instantaneous snapshots of data across space. The second visualization type (view 3 and view 4 in Figure 4) shows time series of data. We first present the visualizations of preprocessed data described in Section 3.1. Views 1–3 may also be used to visualize raw data (non-preprocessed) as shown in Figure 5.

#### 2.1.1. View 1: 2D Spike-Count Rate

The 2D spike-count rate view (Figure 3A, Video 2 in the Supplementary Material) shows instantaneous activity data in separate sub-panels for individual populations. The values in each panel correspond to the instantaneous spike-count rate ν_β_ in one discrete spatiotemporal bin indexed by β = (*l*_*x*_, *l*_*y*_, *k*) in our preprocessed data format (*l*_*x*_ and *l*_*y*_ denote spatial bin indices along the *x*−and *y*−axes, and *k* denotes a temporal bin index). In this format, each spike event is added to the corresponding spatiotemporal bin as described in detail in Section 3.1. The color bar denotes bin values in units of spike counts per second (spikes/s) and is shared between all sub-panels. This view provides a side-by-side comparison of the spatially resolved activity in each individual population. For a larger number of populations (than shown here), it is, however, difficult to relate the activity of one population to another population by visual observation. This problem can however be amended by combining multiple population activities in a single scene.

#### 2.1.2. View 2: 3D Layered Spike-Count Rate

In the 3D layered spike-count rate view (Figure 3B, Video 3 in the Supplementary Material), we combine the activity of all network layers in one 3D-animated scene. The view incorporates the possibility to show also other activity measures, for example the population LFP. The layers in view 2 correspond to the different sub-panels for each population in view 1. Different populations are here assigned unique colors. We chose to illustrate instantaneous spike-count rate ν_β_ by dynamically sized cubic boxes. The box sizes are by default scaled such that their volumes are proportional to ν_β_ at each time step, thus low-activity bins may still be visualized simultaneously with high-activity bins.

View 2 offers multiple possibilities for interactive adaptation of the visualization. As suggested by the information-seeking mantra (Shneiderman, 1996), the user can manually select which part of the data to show, for example by switching on or off individual layers that may occlude visibility of activity in other layers or by setting the horizontal *x*− and *y*−limits of the layers. It is also possible to reduce the opacity of the colored boxes or to scale their side length linearly. The camera can be set either to an orthographic or perspective-corrected projection mode. Dependent on the projection mode, the camera can be moved freely and allows for zooming, panning and rotating the scene. One can easily reset the camera to its default position by the click of a button or select different preset camera positions such as on top or to the side.

The major benefit provided by view 2 over view 1 is the possibility to visually relate the activity in one layer to other layers as all layers are drawn in the same 3D scene. As box volumes are computed from instantaneous spike-count rate values, this view brings the attention of the user to spatial regions of the network with high local activity. While views 1 and 2 offer flexible visualizations of instantaneous activity across space, we next consider scenes capable of showing time-series data.

#### 2.1.3. View 3: Scrolling Spike-Count Rate Plot

The scrolling spike-count rate plot (Figure 4A, Video 4 in the Supplementary Material) is a time-series representation of the data that neglects spatial features of the network and its activity. It shows the time evolution of the total spike-count rate ν_*k*_ (black line), defined as the sum over the spike-count rates of all spatial bins and populations divided by the number of bins, together with the relative rate of each individual population (colored stacked plot). ν_*k*_ is defined as $\nu_{k}\equiv 1/\left(L_{x}L_{y}\right)\cdot \underset{X}{\sum }\underset{l_{x}}{\sum }\underset{l_{y}}{\sum }\nu_{\beta }$ with β = (*l*_*x*_, *l*_*y*_, *k*) and where *L*_*x*_ and *L*_*y*_ denote the number of bins along the *x*− and *y*−axes, and neuronal populations are denoted *X*. The per-population spike-count rates can therefore be inferred by multiplication of the total rate with the fraction of spiking in individual sub-populations. The color coding of each population corresponds to the one used in view 2, but can also be read from the bar to the right. The plot is centered on the current time step (vertical white line indicator) when scrolling through data points in the animation. It allows for interactive change of the width of the visible time window and also permits to manually select or deselect individual populations to be displayed. View 3 provides a temporal overview of the data and allows to identify time intervals of interest, for example, due to an external perturbation.

#### 2.1.4. View 4: Scrolling Spike-Count Rate Iso-Surface Plot

The instantaneous spike-count rates of our example network are time-series activity data with 2D spatial structure. In order to visualize such data without loss of dimensionality, a 3D representation is in general required (unlike for instance view 3). The scrolling spike-count rate iso-surface scene in Figure 4B (and in Video 5 in the Supplementary Material) simultaneously shows the evolution of network activity in space (as in views 1 and 2) and time (as in view 3). The rate iso-value surfaces of each population is rendered using the computer-graphics algorithm “marching cubes” (Lorensen and Cline, 1987). The color coding of the individual populations matches the coding used in views 2 and 3. In terms of user interactivity, the user can set the threshold (isolation) for the surfaces. Furthermore, the user can select which populations to show, vary their opacity level, apply a temporal offset to individual populations, change the width of the time window, and take full control of the viewpoint in the 3D scene as in view 2.

#### 2.1.5. Raw Data Views

In addition to visualizing spatiotemporally binned, preprocessed data, views 1–3 can also be used with raw simulation output files formatted according to the description in Section 3.1. With raw file output, view 1 (Section 2.1.1, Figure 5A) displays for each animation time step a square marker for each spike time $t_{j}^{s}$ at the spatial location (*x*_*j*_, *y*_*j*_) of neuron *j* in population *X*. The square marker color is population specific. Likewise, view 2 (Section 2.1.2, Figure 5B) shows boxes of equal size for each spike event, colored according to population. The view allows, as with precomputed spike-count rates, to show spiking activity in each population in the same scene. We here show a snapshot of the spiking activity in perspective mode, and top-down. The main interactive feature of views 1 and 2 incorporated with raw data files is the option to reduce the neuron density to be displayed. Similar to the visualization of preprocessed data with view 2, individual layers can be activated/deactivated, one can switch between the orthographic and perspective viewing modes, and the camera can be positioned freely. View 3 (Section 2.1.3) applied to raw data is shown in Figure 5C. The temporal bin size of the animation is then equal to the simulation time step *dt* (one spike therefore results in the spike count rate 1/*dt* in units of spikes/*s* for that instant). The total spike count (black line) is summed over all neurons, in contrast to the sum over the population-averaged per-bin rates as in the case of the preprocessed data. The relative spike count per population is shown as a stacked plot normalized by the total amount of spikes in each temporal bin.

### 2.2. VIOLA Use Case

Numerical model development representing a physical system comprises implementation, simulation, analysis as well as comparison, validation, and verification steps. Such model development is important for building hypotheses and aiding interpretation based on experimental data and observations. We here demonstrate how the views described above can be integrated with the development of a spiking point-neuron network model. For this purpose, we can hypothesize that transient external input to a layered spiking point-neuron network model with distance-dependent recurrent connections results in propagating spatiotemporal activity. We wish to assess the spatial extent and temporal duration of the network response to external perturbation and whether or not the unperturbed network state is recovered. In this use case we do this assessment by visual inspection prior to any detailed numerical analysis, focusing on the importance of coordinated multiple views (Wang Baldonado et al., 2000; Roberts, 2007) and Shneiderman's information-seeking mantra (Shneiderman, 1996). For our hypothesis above we will therefore use our implementations of views 1–4 in VIOLA to rapidly analyze our network activity. We show that a combination of the different views is needed to asses the relevant aspects in the data, which is the evoked response to a network perturbation.

The layered point-neuron network illustrated in Figure 1B consists of an excitatory (EX) and an inhibitory (IN) neuronal population plus one stimulus population (STIM). Each neuron is placed randomly within square sheets. EX, IN and STIM units are connected using distance-dependent rules as illustrated in Figure 1A. The connectivity is periodic across boundaries (torus connectivity). The detailed network description is given in Section 3.4. The main simulation output is spike times of individual neurons, neuron locations and a synthetic LFP signal (see Section 3.5 for details). Our initial preprocessing steps and corresponding data formats are described in Section 3.1.

#### 2.2.1. Temporal Features of Evoked Network Activity

**Figure 6:**
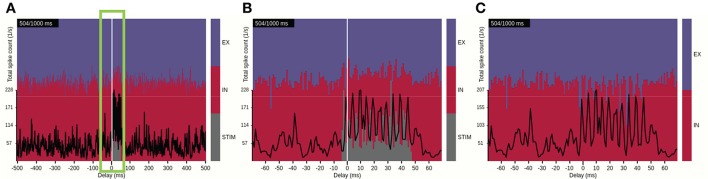
Identifying a time interval of interest with view 3. **(A)** The spike-count rate summed over population-averaged rates and the relative contributions by populations EX, IN, and STIM shown for a time window of ±500 ms around the current time step of the animation. **(B)** Same as **(A)**, but with a narrower time window of ±70 ms (indicated by the green frame in **A**), highlighting the activation of the STIM population and corresponding network response. **(C)** Same as **(B)**, but with the STIM contribution turned off.

We first focus on ongoing activity of the network in the time domain, as provided by view 3. This view implements a scrolling spike-count rate plot which ignores spatial information. Interactive control of the view's time window allows for quick identification of events of interest from the full duration of the simulation (Figure 6A). One such event that is clearly differentiated from other ongoing activity is the activation of the external STIM population at the animation time step of 500 ms. Pausing the animation at 504 ms and zooming in onto the event (Figure 6B) allows for a detailed look on how the total spike-count rate (black trace) increases and oscillates while the stimulus is active, and confirms that the stimulus duration was 50 ms. The color-coded stacked histogram reveals that during stimulus activation a large relative fraction of spike events is contributed by the STIM units (gray), while the relative fraction generated by the recurrently connected EX (blue) and IN (red) units is reduced. We may also conclude that the transient onset of the stimulus results in temporally brief imbalances between excitatory and inhibitory populations in the network as the relative rate of the inhibitory population drops with regular intervals during the stimulation period. The imbalances occur at the stimulus onset and during each period of the resulting network oscillation (from recurrent interactions between excitatory and inhibitory neurons). This network spike-rate imbalance is even more pronounced when the STIM activity is hidden (Figure 6C). We note, however, that the rate balance averaged over the stimulus duration is similar to time-averaged rate balance in the non-perturbed state.

From the visualization we can also infer that the external perturbation to the network does not result in a shifted network state after the stimulus is switched off. Overall rate fluctuations and relative fractions of spike-count rates appear comparable before and after the stimulus period, unlike networks that may display multi-stable patterns of activity (Litwin-Kumar and Doiron, 2012; Miller, 2016) wherein their state can shift from one attractor to another either spontaneously or due to a perturbation.

#### 2.2.2. Spatial Features of Evoked Network Activity

**Figure 7:**
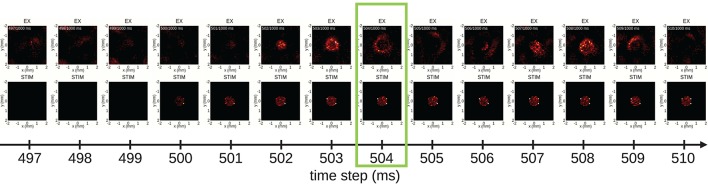
From spontaneous to evoked activity, resolved in time and 2D space. Time frames of populations EX (first row) and STIM (second row) are captured from view 1 every 1 ms. After three frames showing spontaneous activity of population EX, the STIM layer is activated (first visible in the fourth column, at 500 ms), resulting in a repeated pattern of outward spread of activity in the EX layer. The time step highlighted by a green outline (at 504 ms) corresponds to the animation time step in Figure 6.

**Figure 8:**
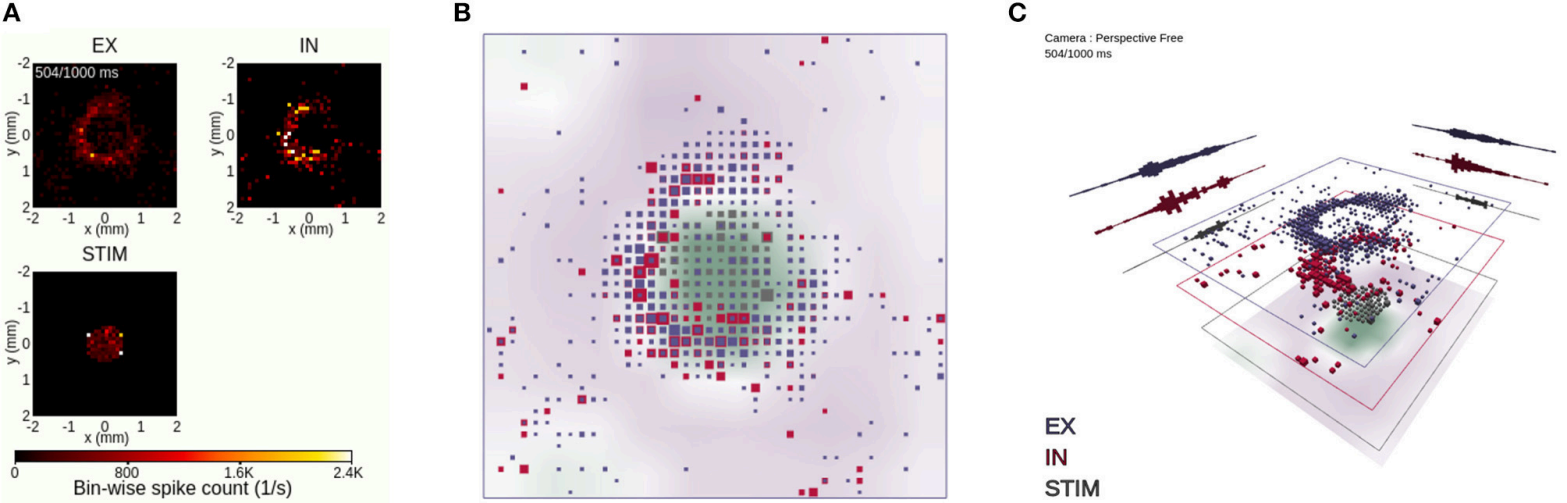
Coordinated views on a temporal snapshot of the neuronal activity. **(A)** Instantaneous spike-count rates in layers EX, IN and STIM using view 1. The animation time step of 504 ms is identical to the one in Figures 6 and 7 in this and subsequent panels. **(B)** Orthographic top-down view onto stacked population layers and LFP image plot with view 2. **(C)** Perspective view with large layer separation, including summed spike counts projected toward the layer edges in view 2.

**Figure 9:**
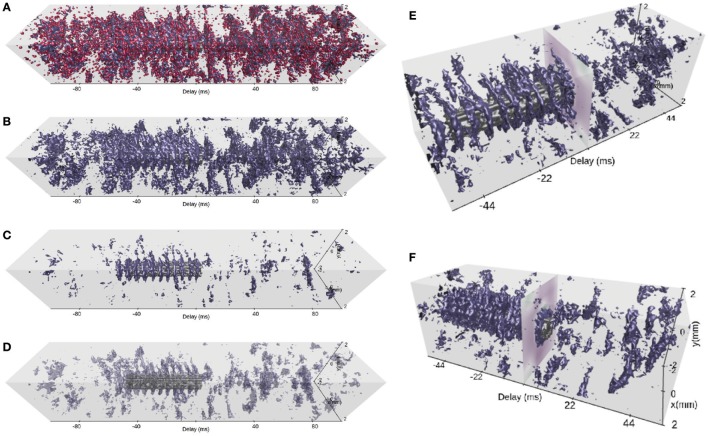
Spatiotemporally resolved activity. **(A)** Spike-count rates across time and space of populations EX (blue), IN (red), and STIM (gray) shown with view 4 for a time window of ±100 ms around the present time step of the animation. The isolation threshold is set to a rate of 100 spikes/s. The animation time step is identical to the one in Figures 6–8 in this and subsequent panels. **(B)** Same as **(A)**, but with IN activity turned off. **(C)** Same as **(B)**, but with an increased isolation threshold of 360 spikes per second. **(D)** Same as **(C)**, but with reduced opacity of layer EX activity and an isolation threshold of 195 spikes/s. **(E)** A narrower time window (±55 ms) and shifted camera position (isolation threshold of 195 spikes/s). The image plot at a delay of 0 ms shows the synthesized LFP signal across space. **(F)** Same as **(E)**, but with the camera position rotated around the vertical *z*−axis.

Having identified a time segment of particular interest (the stimulus duration), we next exploit view 1, the 2D spike-count rate view, and focus on spatial aspects of the evoked network activity. Figure 7 shows a series of snapshots from the instantaneous spike-count rate animation across space for the EX (top row) and the STIM (bottom row) layers. Snapshots are shown for successive bins of width Δ*t*. The first three columns in Figure 7 show spontaneous activity of the EX units. Thereafter the STIM population is switched on, as seen in the fourth column of the bottom row. The activity of the STIM layer is by construction confined to a circle at the center of the network. As the activity of the *N*_STIM_ units in the STIM layer is governed by Poisson processes with rate expectations ν_STIM_, its spike intensity remains fairly constant (except for the bin at 500 ms as the time bin is centered on the time step). In layer EX, the stimulus elicits an increase in activity spreading outwards from the center. This response dies out after a few milliseconds due to recurrent inhibition, but reoccurs regularly as reflected by the oscillatory behavior observed in Figure 6B. The time step at 504 ms highlighted by the green outline is the same as in Figure 7 and latter Figures 8 and 9.

#### 2.2.3. 2D and 3D Views of Spatial Activity

In order to relate the spatial relationship between activity in individual populations, we compare in Figure 8 three different layer-wise animations of neuronal activity. View 1 (Figure 8A) shows individual 2D image plots for the spike-count rates per population, with a shared color bar coding for instantaneous spike-count rate values. This view offers an accurate spatial representation of network activity in temporal bins of width Δ*t*, showcasing the locality of the STIM layer activity and the wider spread of evoked activity in the EX and IN layers. This view does not, however, offer interactive features except time control of the animation (shared with views 2–4) and global scaling of the color-value mapping (sensitivity control, shared with views 2 and 3).

The 3D-scene provided by view 2 adds additional interactive features and incorporates the layer-resolved data of view 1 in one animation (Figures 8B,C). Figures 8B,C show the same temporal snapshot of activity as in Figure 8A. The view shows also the spatial variation of the LFP signal that we synthesized from network activity. The LFP signal, here shown as image plot with a color-coding reflecting its magnitude and sign, is more difficult to relate to the ongoing activity, as it is inherently a signal driven by past spiking activity (resulting of delayed synaptic activation on postsynaptic neurons from spiking activity in presynaptic neurons, cf. Section 3.5). We then compare rate values of one spatial bin and one population to other spatial locations and other populations through their different color codings and cube sizes. An observation is that activity in the EX and IN layers are typically confined within the same spatial region of the network, while a larger fraction of the network is quiescent at the time. This observation can for example explain high variability in interspike-intervals of individual neurons (Keane and Gong, 2015), as neurons may fire frequently while fronts of activity spread across the network and remain quiet until the next burst of activity.

In terms of using interactive features offered in view 2, we turn off the orthographic mode of Figure 8B and go back to its default 3D perspective in Figure 8C. We also rotate the viewpoint in order to directly focus on highly active parts of the network. Furthermore, the different layers of the network and LFP are offset vertically, with dynamic projections of the sum of spiking activity across each respective spatial axis for each network layer. From this setup of view 2, we can better infer the activity in each individual layer, including that of the LFP layer, without switching off individual layers.

#### 2.2.4. Spatiotemporally Resolved Network Activity

We finally investigate network activity in space and time using the 3D scene provided by view 4. Similar to the scrolling spike-count rate plot of view 3, view 4 allows full control of the time axis. The activity of all populations EX, IN and STIM is displayed for a wide (200 ms) temporal segment using red, blue and gray iso-surfaces, respectively, in Figure 9A. We have centered the current time step (at 504 ms) on the evoked activity in the STIM layer (highlighted in Figure 7). It is already possible to identify activity patterns confined in space and time. However, it remains difficult to assess how spontaneous network activity changes in response to the stimulus due to occlusion of one surface by another, an inherent issue with multiple solid surfaces. In Figure 9B we therefore hide the activity of the IN layer and focus on the activity in the EX layer. The surfaces correspond to the bin-wise instantaneous spike-count rates at an isolation threshold of 100 spikes/s. Increasing this threshold to 360 spikes/s (Figure 9C) reveals that regular bursts of high rates occur at the center of the layer, in the period when the STIM layer is activated. In the other views, these bursts may be seen as rate oscillations (Figure 6) or pulsating spatial activity (Figures 7 and 8). We here show that the attenuation of activity radiating outward from the center is rather strong.

Using view 4, both the oscillation frequency and the outward spread of activity in the EX population can be assessed. We highlight the STIM activity by reducing the opacity of the EX surfaces in Figure 9D. This reduces occlusion problems present with multiple overlapping opaque surfaces, and thus allows relating the activity in these two populations to one another. A smaller time segment of the scene is shown in panels E and F where we also demonstrate different camera positions. Rotating the camera allows us to observe the synthesized LFP signal at the current time step, and the corresponding network interactions resulting in a strong LFP fluctuation. We also observe the temporal offset between stimulus onset and a response in the EX activity as shown in Figure 9F.

In contrast to the previously discussed applications of views 1–3, the 3D-scene of view 4 allows to relate both temporal and spatial aspects of the spiking activity of different neuronal populations and the LFP signal to one another. With this view, we can get an overview of a large time segment and several populations and then use its incorporated interactive features in order to explore the network activity under influence of the stimulus. The focus of this view lies on highlighting qualitatively interesting features of the data on spatiotemporal scales such as the oscillating activity of EX population surrounding the STIM location (as in Figure 9C) or the temporal offset between STIM and EX seen in Figure 9F. Views 1 and 3, however, better resolve quantitative rate values or temporal offsets, respectively, than views 2 and 4.

## 3. Methods

### 3.1. Data Formats

The data we consider for visualization are sequences *S*_*j*_ of spike times $t_{j}=\underset{s\in S_{j}}{\sum }\delta \left(t_{j}^{s}\right)$ of a neuronal unit *j* ∈ *X* located at coordinate (*x*_*j*_, *y*_*j*_), where *X* denotes a neuronal population of size *N*_*X*_. Individual spike times $t_{j}^{s}$ are constrained to a discrete grid *n* · *dt* for *n* ∈ {0, 1, 2, …, *n*_steps_ − 1}, where *dt* is the time-resolution of spike acquisition and *n*_steps_ the number of time steps in the acquisition period *T*. We assume that the raw spike data to be visualized is available in two pure text files per population *X*. The first file contains two columns with values separated by a white space. Its first column contains integer numbers representing “global neuron identifiers” (neuron IDs) *j*, while the second column contains corresponding spike times $t_{j}^{s}$ in units of ms. This data format, first introduced for experimental data and reviewed in Rostami et al. (2017), is the default output format for spike data of the neuronal network simulator NEST (Kunkel et al., 2017). While the floating point data type is sufficient for displays and the computation of single-neuron and population spike rates, the format is only safe for correlation analysis if the time step is a power of two (Morrison et al., 2007, A.2). The latter guarantees that spike times have a representation in the data type. An alternative is to use the original definition of the format and denote spike times by the integers *n*, thus expressing time in units of the resolution of the grid. The second file contains three space-separated columns. Its first column contains unit IDs *j*, while columns two and three contain the corresponding coordinates *x*_*j*_ and *y*_*j*_ in units of mm. NEST internally represents networks as a graph where edges denote connections. Neurons cannot be interrogated for their location and are only identified by their ID, thus the information on the location must be defined and stored explicitly.

We consider another text-based data format for the visualization of spike data that are preprocessed by a temporal and spatial binning procedure. For the temporal binning we define a temporal bin size Δ*t* as an integer multiple of the acquisition time resolution *dt*. For spatial binning of neuron positions along the *x*− and *y*−axes we define the bin widths Δ*l*. The third spatial dimension (*z*−axis) is ignored. Assuming an acquisition period *T* and the side length *L* of the centered square network domain, the number of temporal bins is *K* = *T*/Δ*t* and the numbers of spatial bins along each axis {*L*_*x*_, *L*_*y*_} = *L*/Δ*l*. A spatiotemporal bin is indexed by the length-three tuple of indices β = (*l*_*x*_ ∈ {0, 1, …, *L*_*x*_ − 1}, *l*_*y*_ ∈ {0, 1, …, *L*_*y*_ − 1}, *k* ∈ {0, 1, …, *K* − 1}), spanning *x* ∈ [*l*_*x*_Δ*l* − *L*/2, (*l*_*x*_ + 1)Δ*l* − *L*/2), *y* ∈ [*l*_*y*_Δ*l* − *L*/2, (*l*_*y*_ + 1)Δ*l* − *L*/2) and *t* ∈ [*k*Δ*t*, (*k* + 1)Δ *t*). In each spatiotemporal bin, we sum for every population *X* the number of spike events and divide by the temporal bin size Δ*t*. We refer to this measure as the instantaneous spike-count rate ν_β_ in units of 1/s. The preprocessed data is contained in one single file per population with four space-separated columns. Indices *l*_*x*_, *l*_*y*_, and *k* for each spatiotemporal bin are put in columns one, two, and three, respectively, while the 4th column contains the corresponding rate value. Rows are ordered in iteration running order according to *k* ∈ [0, 1, …, *K* − 1] over all *l*_*x*_ ∈ [0, 1, …, *L*_*x*_ − 1] and finally over all *l*_*y*_ ∈ [0, 1, …, *L*_*y*_ − 1]. Row entries where ν_β_ = 0 are not written. The same data format is used to represent the evolution of spatially organized analog data with spatial resolution Δ*l*^ϕ^. The unit of the data depends on the actual measure, for example mV in case of the LFP.

### 3.2. Reference Implementation

**Figure 10:**
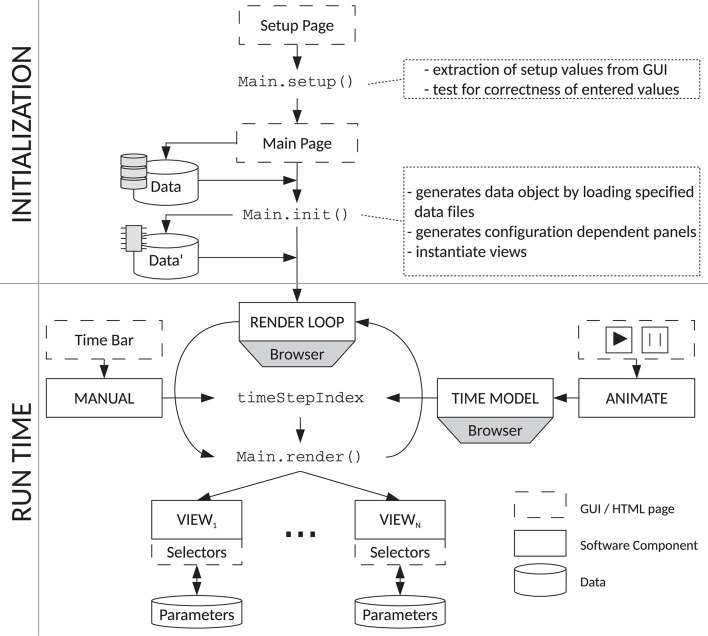
Flow chart of VIOLA's components. VIOLA incorporates two main parts: “initialization” (top) and “run time” (bottom). The initialization procedure defines a Setup and Main Panel in VIOLA's GUI and the corresponding Main.setup() and Main.init() functions. The Main.setup() function is used for setting initial values, while the Main.init() function allows for loading datasets with parameters that depend on setup values. The Main.init() function also instantiates the different views in the application. The run-time component of VIOLA uses a rendering loop and time model provided by the web browser. The time model is needed to synchronize the rendering of each view, such that at all times each view shows the same time step of the data and thus compensates for different redering times of the views. By default, the timeStepIndex in the rendering loop is automatically updated by the browser (using the time model), but can also be set by the user, e.g., by a slider widget. The browser time model is controlled using the “animate” widget, while a time bar is used for “manual” time selection. Each update of the timeStepIndex triggers an execution of the Main.render() method and a corresponding update of all views. Parameters for the different views can be modified during run time as each view offers its individual input widgets.

We have made implementations of the visualization types discussed throughout this paper available in the tool VIOLA (VIsualization Of Layer Activity). Figure 10 illustrates the web-based JavaScript framework integrating the different visualizations we refer to as “views.” A central class named Main carries out the initialization and coordination of the views. The Graphical User Interface (GUI) is comprised of two main components, the Setup Panel and the Main Panel. The Main Panel also serves as a container for the views. The Setup Panel is the first entity presented to the user when the application is opened in a web browser. It serves mainly to specify the data types to be loaded and the basic data features (spatial dimensionality, time resolution) and visualization features such as the colors for each neuron population. Parameters can be set manually or be loaded from configuration files, for example specifying whether to load raw or preprocessed data. These configuration files are JavaScript Object Notation^8^ (JSON) files specifying the format of the loaded data, file names, and preset values for the visualizations. After confirming the entered information, the Main.setup() function extracts the entries and provides them globally to the other components. The Main Panel shown afterwards is used to load the input data files from the local file system using JavaScript FileReader, which includes setting up the internal data structure giving a coherent access to the data to be visualized. The Main.init() function then initializes the rendering loop, which is built into the web browser and controls the rendering of the various views. The Main.render() method is executed periodically by built-in functionality of the browser, which is further used to synchronize the rendering of all views. This is necessary as the rendering of an individual time step needs different amount of time per view. For example, rendering a complex 3D scene is slower (because it need more computational resources) than rendering a less complex 2D plot. All views must have finished rendering before the next rendering step is triggered. Each rendering call is triggered by updating the global timeStepIndex through the browser. The update of the timeStepIndex calls the rendering loop, which redirects the rendering call to all views. For rendering the data, all views access the loaded simulation data structure as part of the Main object.

The timeStepIndex variable can be controlled in two modes. First, it can be manually set by the user via a slider widget. The slider widget enables the user to scroll manually along the time axis, thus offering a simple navigation through the time series. Second, the user can start an animation of the loaded data set by pressing a start button. This triggers a periodic update of the timeStepIndex. As both manual and periodical updates of the timeStepIndex trigger the same downstream functionality for updating the views, manual navigation through the time series can be combined with automatic updates of the timeStepIndex. In case the animation is running, manual intervention by the user overrides the periodic update of the timeStepIndex, such that the shown data item (time step) corresponds to that manually selected one. The animation is continued from this manually selected time step.

All views offer view-specific selectors for visualization parameters. These parameters are read in and used in each render call.

### 3.3. 2D and 3D View Implementations

This section describes the visualization algorithms used with the different view implementations in VIOLA as introduced throughout Section 2. As the output data produced by the simulation are scalars organized on a regular grid (related to the neuron's position), visualization methods applicable to scalar data are employed as well as standard chart types (Hansen and Johnson, 2011). View 1 implements a standard image plot in which the color of each spatial bin represents a spike-count rate value. The display maps a binned measure of neuronal activity on a color by using a lookup table ranging from black to white over red and yellow (often referred to as “red hot” or “white hot” lookup table). We select this lookup table as it is widely used and offers (if non-linear interpolated) equidistant colors according to the CIE L*a*b* color space [EN ISO 11664-4 from 1976]^9^. This color space is designed to represent equidistant colors according to human perception: a color twice as light in CIE L*a*b* space is also perceived twice as light by a human user (Fairchild, 2013). Such a heat map (Spence, 2014) facilitates the representation of the spatial structure of the data.

In the 3D visualization of view 2, VIOLA also implements a geometrical mapping of activity data to a cube's edge length, resulting in a cubic mapping of the single scalar value representing activity at each time step. The default scaling of the cube's edge length is such that the cube's volume is proportional to the data value in each bin.

In view 3, the concept of a stacked bar chart supports a global perspective on the simulated model (Spence, 2014). Individual populations in the model are color-coded to be separable in the bar chart. An additional line graph added on top of the bars shows the total spike-count rate as reference to the global activity.

For view 4, in order to support the visual interpretation of time series of spatially organized activity data, the 2D-organized activity data (considering the neurons in one layer) are extracted along the time axis resulting in a regularly structured 3D volume of scalar values. Through contouring, partial sub-volumes with a certain minimum threshold of activity get extracted and rendered as geometry. By means of a selected iso-value *I*_th_ such geometry gets extracted by applying the marching cubes algorithm for implicit volume rendering (Lorensen and Cline, 1987). For extraction of the geometry, the algorithm assumes that each data point of the data set is mapped onto a vertex (corner point) of a regular 3D grid, which can be subdivided into cells delimited by eight neighboring vertices each. Then, the algorithm calculates for each vertex of a cell whether the associated data value of the considered vertex lies inside or outside of the contour defined by the iso-value *I*_th_ by comparing the data value with the iso-value. If the data value of a vertex is smaller than the iso-value, the vertex is assumed to lay inside of the contour. For each possible combination of inside/outside states of the vertices of a cell, the topology of the contour for each cell gets extracted from a table by calculating a representative index. This table holds all possible topological states of a cell, which are constructed under the assumption that there is an infinite number of possibilities of how a contour can pass a cell (for more details, please refer to, for example Hansen and Johnson, 2011, Chapter 1). Finally, the exact position of the contour gets calculated by interpolation along the cell's edges.

Views 2, 3, and 4 all use the same color coding to identify the different neuronal populations. The implementation of the algorithms and views uses native JavaScript. The 2D rendering routine uses the HTML5 canvas element. The browser rendering engine supports HTML5 and especially the functionality of the canvas element, therefore no external libraries are required. 3D renderings relies on the three.js^10^ wrapper for WebGL content which is natively supported by the engine of modern browsers. Node.js^11^ facilitates the communication between views and the GUI for control.

### 3.4. Network Description

The example network is based on an implementation of a random balanced network (Brunel, 2000) which is part of NEST as an example (brunel_alpha_nest.py in NEST 2.12.0 by Kunkel et al., 2017). The model is expressed using PyNEST (Eppler et al., 2009) in Python^12^. The network consists of *N*_EX_ excitatory and *N*_IN_ inhibitory spiking point-neurons which are sparsely connected with connection probability *c*. Neurons have fixed in-degrees of *cN*_EX_ excitatory and *cN*_IN_ inhibitory incoming synapses with weights *g*_*Y*, EX_*J* and *g*_*Y*, IN_*J*, respectively, with *Y* ∈ {EX, IN}. The integrate-and-fire model neurons are connected using static, current-based synapses with an alpha-shaped time course (NEST neuron model: iaf_psc_alpha). The intrinsic neuron parameters are identical for both neuron types. In addition to the recurrent connections, each neuron receives uncorrelated, external excitatory input from a Poisson process of a fixed rate ν_ext_ = ην_θ_, where η denotes the external rate relative to the threshold rate ν_θ_ which is defined as ν_θ_ = (*V*_θ_ − *E*_L_)*C*_m_/(exp(1)*J*τ_m_τ_s_). The threshold rate is the hypothetical external rate needed to bring the average membrane potential of a neuron to threshold *V*_θ_ (in the absence of an actual spiking mechanism). *E*_L_ denotes the resting potential, *C*_m_ the membrane capacitance, τ_m_ the membrane time constant and τ_s_ the postsynaptic current time constant.

Unlike the original network implementation which has no spatial information, we here place neurons randomly on a square 2D sheet with side lengths *L*. The connection probability between a presynaptic neuron *j* and postsynaptic neuron *i* decays with increasing horizontal distance *r*_*ij*_ (using periodic boundary conditions) while we preserve the in-degrees (number of incoming connections). A Gaussian-shaped profile *p*_*YX*_(*r*_*ij*_) is used with a standard deviation of σ_*YX*_ with *X, Y* ∈ {EX, IN}. We use ϵ_*YX*_(*r*_*ij*_) to describe the distance-dependent connectivity profile assuming that the in-degree is preserved. The transmission delay function *d*_*YX*_(*r*_*ij*_) has a linear distance dependency with an offset $d_{YX}^{0}$ and a conduction velocity *v*_*YX*_.

In addition to the stationary external input to each population, the network receives a spatially confined transient input with a duration *t*_STIM_. The input is provided by a size *N*_STIM_ population of parrot neurons (NEST's parrot_neuron devices), positioned inside a circle of radius *R*_STIM_ around (*x, y*) = (0, 0). Parrot neurons simply repeat input spike events as output spike events. Each parrot neuron receives input from a Poisson process with a rate expectation of ν_STIM_ and connected to *K*_STIM_ neurons of the EX population inside a connection mask radius *R* from the parrot-neuron location. The Poisson input starts at *T*_STIM_ and consequently the STIM units become active after a delay of *d*_STIM_.

Table 1 summarizes the network description with model and simulation parameters listed in Tables 2A,B. The original parameters for the EX–IN network in the NEST example are modified for this VIOLA use case demonstration bringing the network in a state with spatially confined network activity. For this we increase the network's neuron count, reduce the ratio of inhibitory to excitatory weights *g*_*Y*, IN_ and the membrane capacitance *C*_m_, while the postsynaptic amplitude *J* is increased. The parameter *J* is originally defined in units of mV, but is here re-defined in units of pA. Finally, the fixed conduction delay is replaced by a distance-dependent one.

**Table 1:** Description of the network model following the guidelines of Nordlie et al. (2009).

A: MODEL SUMMARY	
Populations	Three: excitatory EX, inhibitory IN, external stimulus STIM
Topology	EX/IN: random neuron positions on square domain of size *L* × *L*; STIM: random neuron positions inside a circle with radius *R*_STIM_ at the center of the domain; periodic boundary conditions
Connectivity	Random (EX/IN: convergent, fixed in-degree; STIM: divergent, fixed out-degree) connections described by distance-dependent probability kernels and cut-off masks
Neuron model	EX/IN: leaky integrate-and-fire (LIF), fixed threshold, absolute refractory time; STIM: parrot
Synapse model	Static weights, EX/IN: alpha-shaped postsynaptic currents, distance-dependent delays
Input	Independent fixed-rate Poisson spike trains to all neurons
Measurement	Spike activity
B: NETWORK MODEL	
Subthreshold dynamics	EX/IN: If $t>t^{*}+\tau_{\text{ref}}$ $\frac{\text{d}V}{\text{d}t}=-\frac{V-E_{L}}{\tau_{\text{m}}}+\frac{I_{\text{syn}}\left(t\right)}{C_{\text{m}}}$ $I_{\text{syn}}\left(t\right)=\underset{j}{\sum }J_{j}\alpha \left(t-t_{j}^{*}-d_{j}\right)$ with connection strength *J*_*j*_, presynaptic spike time $t_{j}^{*}$ and conduction delay *d*_*j*_ $\alpha \left(t\right)=\frac{t}{\tau_{\text{s}}}{\text{e}}^{1-t/\tau_{\text{s}}}\Theta \left(t\right)$ with Heaviside function Θ else *V*(*t*) = *V*_reset_
Spiking	If *V*(*t*−) < *V*_θ_∧*V*(*t*+)≥*V*_θ_ 1. set t^*^ = *t* 2. emit spike with timestamp t^*^ 3. reset *V*(*t*) = *V*_reset_
Distance-dependent connectivity	Neuronal units *j*∈*X* at location (*x*_*j*_, *y*_*j*_) and *i*∈*Y* at (*x*_*i*_, *y*_*i*_) in pre- and postsynaptic populations *X* and *Y*, respectively. Distance between units *i* and *j*: $r_{ij}=\sqrt{{\left(x_{i}-x_{j}\right)}^{2}+{\left(y_{i}-y_{j}\right)}^{2}}$ Gaussian kernel for connection probability: $p_{YX}\left(r_{ij}\right)={\text{e}}^{-r_{ij}^{2}/2\sigma_{YX}^{2}}$ *R* is the radius of a cut-off mask. Transmission delay function: $d_{YX}\left(r_{ij}\right)=d_{YX}^{0}+r_{ij}/v_{YX}$

**Table 2:** Simulation, network, and preprocessing parameters.

**Symbol**	**Value**	**Description**
**A: GLOBAL SIMULATION PARAMETERS**		
*T*_sim_	1, 500 ms	Simulation duration
*dt*	0.1 ms	Temporal resolution
*T*_trans_	500 ms	Startup transient
*T*_STIM_	999 ms	Start time of Poisson input to STIM
*t*_STIM_	50 ms	Duration of STIM onset
**B: POINT-NEURON NETWORK**		
**Populations and external input**		
*X*	EX, IN, STIM	Name
*N*_*X*_		Population size:
	20, 000	*X* = EX
	5, 000	*X* = IN
	975	*X* = STIM
*L*	4 mm	Extent length
η	2	External rate relative to threshold rate for *X* ∈ {EX, IN}
*R*_STIM_	0.5 mm	Radius of circle around (0, 0) for locations of STIM
ν_STIM_	300 *Hz*	External rate to each STIM neuron
**Connection Parameters**		
*c*	0.1	Connection probability for recurrent connections between EX and IN
*J*	40 *pA*	Reference synaptic strength. All synapse weights are measured in units of *J*.
*g*_*YX*_		Relative synaptic strengths:
	1	*X* = EX, *Y* ∈ {EX, IN}
	−4.5	*X* = IN, *Y* ∈ {EX, IN}
	1	*X* = STIM, *Y* = EX
*R*	0.1 mm	Radius of cut-off mask for X = STIM, Y = EX
*K*_STIM_	300	Number of connections per STIM neuron
σ_*YX*_		Standard deviation of Gaussian kernel:
	0.3 mm	*X, Y* ∈ {EX, IN}
$d_{YX}^{0}$		Delay offset:
	0.5 ms	*X, Y* ∈ {EX, IN}
	0.5 ms	*X* = STIM, *Y* = EX
*v*_*YX*_		Conduction velocity:
	2 m/*s*	*X, Y* ∈ {EX, IN}
	−	*X* = STIM, *Y* = EX
*d*_STIM_	0.5 ms	Delay from Poisson input to STIM
**Neuron model**		
*C*_m_	100 *pF*	Membrane capacitance
τ_m_	20 ms	Membrane time constant
*E*_L_	0 *mV*	Resting potential
*V*_θ_	20 *mV*	Firing threshold
*V*_reset_	0 *mV*	Reset potential
τ_ref_	2 ms	Absolute refractory period
τ_s_	0.5 ms	Postsynaptic current time constant
**C: PREPROCESSING**		
Δ*t*	1 ms	Temporal bin size
Δ*l*	0.1 mm	Spatial bin size

The data sets result from simulations of duration *T*_sim_ with a temporal resolution of *dt*. We discard the startup transient period *T*_trans_ and record all spike times from all neurons. The unprocessed spike times together with the corresponding neuron positions are considered as raw output. The temporal and spatial bin sizes used for preprocessing, Δ*t* and Δ*l* respectively, are given in Table 2C.

### 3.5. LFP Predictions

#### 3.5.1. Generation of LFP-like Data

The local field potential (LFP) is, due to its relative ease of measurement, a common measure of neuronal activity (Buzsáki et al., 2012; Einevoll et al., 2013). The LFP is, in general, assumed to reflect synaptic activity and correlations of a large number of neurons in vicinity of the recording electrodes (Kajikawa and Schroeder, 2011; Lindén et al., 2011; Łeski et al., 2013). For the purpose of demonstrating VIOLA's functionality, we synthesize LFP signals from network activity assuming a linear network-population spike to LFP relationship $H_{X}\equiv H_{X}\left(\vec{\Delta },\tau \right)$ derived using a biophysical model. In this relationship, $\vec{\Delta }$ denotes the displacement between the center of a spatial bin and an electrode contact point γ at **r**_γ_, and τ the time relative to a presynaptic spike event (“lag”). Assuming linearity and homogeneous spike-LFP responses of individual presynaptic neurons located within the same bin of width Δ*l*^ϕ^ indexed by $b=\left(l_{x}^{\phi },l_{y}^{\phi }\right)$ (see Section 3.1), the signal ϕ_*X*_ at one contact γ of one population *X* is then given by

(1) $$\begin{array}{l} \phi_{X}\left({\text{r}}_{\gamma },t\right)=\underset{b}{\sum }\left(\left(\underset{s}{\sum }\delta \left(t_{b}^{s}\right)\right)*H_{X}\right)\left({\text{r}}_{\gamma },t\right)\;. \end{array}$$

Here, the term $\underset{s}{\sum }\delta \left(t_{b}^{s}\right)$ represents a series of spike times $t_{b}^{s}$ of all presynaptic neurons in a bin *b* where δ denotes the Dirac delta function, and ^*^ a convolution. As contributions of different populations *X* sum linearly, the total signal at each contact is

(2) $$\begin{array}{l} \phi \left({\text{r}}_{\gamma },t\right)=\underset{X}{\sum }\phi_{X}\left({\text{r}}_{\gamma },t\right)\;. \end{array}$$

Point-like neurons (as used in our network model) can not generate an extracellular potential, as all in- and outgoing currents sum to zero at the point's location (due to conservation of charge). As in Hagen et al. (2016) we assume that spatially extended (morphologically detailed) neurons and corresponding multicompartment models in combination with an electrostatic forward model are required to compute a biophysically meaningful LFP signal. To compute the LFP, we here derive for each presynaptic population *X* ∈ {EX, IN, STIM} the phenomenological mapping $H_{X}\left(\vec{\Delta },\tau \right)$ between a presynaptic spike event time $t_{b}^{s}$ occurring in a spatial bin indexed by *b* to the extracellular potential.

#### 3.5.2. Measurement Sites

The electrode contact point locations are defined at the center of each spatial bin as ${\text{r}}_{\gamma }=\left(\left(l_{x}^{\phi }+1/2\right)\Delta l^{\phi }-L/2,\left(l_{y}^{\phi }+1/2\right)\Delta l^{\phi }-L/2,0\right)$.

#### 3.5.3. Multicompartment Model

We define a ball-and-stick type multicompartment model neuron with morphological features and passive parameters derived from the network's LIF neuron description (membrane capacitance *C*_m_, membrane time constant τ_m_, passive leak reversal potential *E*_L_). Assuming a homogeneous specific membrane capacitance *c*_m_ (capacitance per membrane area) and axial resistivity *r*_a_ (resistance times length unit), we choose the dendritic stick length *L*_dend_ and radius *r*_dend_ as follows: To preserve the total capacity of the point neuron (and equivalent surface area), we compute the corresponding soma radius as $r_{\text{soma}}=\sqrt{\frac{C_{\text{m}}}{4\pi c_{\text{m}}}-\frac{r_{\text{dend}}L_{\text{dend}}}{2}}$. We define the passive leak conductivity as *g*_L_ = *c*_m_/τ_m_ and leak reversal potential as *E*_L_. For these calculations we choose *c*_m_, *r*_a_, *L*_dend_ and *r*_dend_ values as given in Table 3, resulting in *r*_soma_ ≈ 13.1 μm. The compact ball-like soma is treated as a single segment, while the elongated dendrite is split into *n*_dend_ = 11 segments of equal length. The center of the soma segment is set to **r** = (0, 0, 0), and the dendritic stick is aligned in the positive direction along the vertical *z*−axis.

**Table 3:** Parameters for prediction of LFP signals.

**Symbol**	**Value**	**Description**
**SIMPLIFIED LFP MODEL PARAMETERS**		
*c*_m_	1 μ*F*/*cm*^2^	Specific membrane capacitance
*r*_a_	150 Ω*cm*	Axial resistivity
*L*_dend_	500 μm	Dendritic stick length
*r*_dend_	2.5 μm	Dendritic stick radius
*n*_dend_	11	Dendritic stick number of segments
*r*_soma_	13.1 μm	Derived soma segment radius
τ^*s*^	25 ms	Synapse activation time
Δ*l*^ϕ^	400 μm	Electrode separation, spatial bin width
σ_e_	0.3 *S*/m	Extracellular conductivity

#### 3.5.4. Synapse Model

For LFP predictions we use the same current-based synapse model as in the network, defining the postsynaptic input current of a single presynaptic spike event as $I_{ij}\left(t\right)=J_{YX}\cdot \left(t-t_{j}^{s}-d_{ij}\right)/\tau_{\text{syn}}\exp\left(1-\left(t-t_{j}^{s}-d_{ij}\right)/\tau_{\text{syn}}\right)\Theta \left(t-t_{j}^{s}-d_{ij}\right)$, where *J*_*YX*_ denotes the connection-specific postsynaptic current amplitude as in the network, $t_{j}^{s}$ the presynaptic spike time, *d*_*ij*_ = *d*_*YX*_(*r*_*ij*_) the conduction delay between presynaptic cell *j* and postsynaptic cell *i* and Θ the Heaviside step function. As we initially ignore delays and network spike times we set *d*_*ij*_ = 0 and $t_{j}^{s}=\tau^{s}$.

#### 3.5.5. Synaptic Connectivity

For outgoing connections of the excitatory populations *X* ∈ {EX, STIM} we distribute synaptic input currents evenly along the entire length of the dendritic stick, while for outgoing connections of the inhibitory population *X* = IN all synaptic input currents are assumed to be evenly distributed on the ball-like soma.

#### 3.5.6. Electrostatic Forward Model

As described in detail in Lindén et al. (2014), we assume an extracellular conductive medium that is linear (frequency independent), isotropic (identical in all directions), homogeneous (identical in all positions), and ohmic (linear relationship between current density and electric potential), as represented by the scalar conductivity σ_e_ (cf. Table 3 for values). From the linearity of Maxwell's equations, contributions to the extracellular potential from different current sources sum linearly. Here, these current sources are transmembrane currents (summed over resistive, capacitive and synaptic currents). In the presently used volume conduction theory, the electric potential in location **r**_γ_ from a point current with magnitude *I*(*t*) in location **r**_0_ is

(3) $$\begin{array}{l} \phi_{\text{point}}\left({\text{r}}_{\gamma },t\right)=\frac{1}{4\pi \sigma_{\text{e}}}\frac{I\left(t\right)}{\left|{\text{r}}_{\gamma }-{\text{r}}_{0}\right|}\;. \end{array}$$

This relation is also valid for a sphere current source (i.e., our ball soma) centered at **r**_0_ with total transmembrane current *I*_m,soma_ and radius *r*_sphere_ when |**r**_γ_ − **r**_0_| ≥ *r*_sphere_. Thus

(4) $$\begin{array}{l} \phi_{\text{soma}}\left({\text{r}}_{\gamma },t\right)=\frac{1}{4\pi \sigma_{\text{e}}}\frac{I_{\text{m},\text{soma}}\left(t\right)}{\left|{\text{r}}_{\gamma }-{\text{r}}_{\text{soma}}\right|}\;. \end{array}$$

The elongated dendritic segments are treated as “line sources,” obtained by integrating the point-source formula along the central axis of the segments (Holt and Koch, 1999; Lindén et al., 2014):

(5) $$\begin{array}{l} \phi_{\text{dend}}\left({\text{r}}_{\gamma },t\right)=\frac{1}{4\pi \sigma_{\text{e}}}\overset{n_{\text{dend}}}{\underset{u=1}{\sum }}I_{\text{m},u}\left(t\right)\int \frac{d{\text{r}}_{u}}{\left|{\text{r}}_{\gamma }-{\text{r}}_{u}\right|}\;. \end{array}$$

The total extracellular potential from somatic and dendritic sources is then

(6) $$\begin{array}{l} \phi \left({\text{r}}_{\gamma },t\right)=\phi_{\text{soma}}\left({\text{r}}_{\gamma },t\right)+\phi_{\text{dend}}\left({\text{r}}_{\gamma },t\right)\;. \end{array}$$

Our calculations of extracellular potentials rely on the Python package LFPy^13^ (Lindén et al., 2014; Hagen et al., 2018). The tool implements the above forward-model formalism for extracellular potentials, and uses the NEURON simulation environment (Carnevale and Hines, 2006) to compute transmembrane currents *I*_m_(*t*) of multicompartment neuron models. As singularities may occur in the limit |**r**_γ_ − **r**_*u*_| → 0, the minimum distance between sources and measurement locations was set equal to the somatic or dendritic segment radius.

#### 3.5.7. Prediction of Spike-LFP Relationship

We here describe the calculation of the linear spike-LFP relationships $H_{X}\left(\vec{\Delta },\tau \right)$ which we use to construct an LFP-like signal from spatially binned network activity. While Hagen et al. (2016) present a hybrid scheme to compute extracellular potentials from point-neuron network activity, and incorporated the biophysics-based forward model summarized above, this hybrid scheme is not adapted to laminar point-neuron networks with distance-dependent connections. We therefore construct a simpler and numerically much less demanding method inspired by the hybrid scheme, that still encompasses the governing biophysics underlying the generation of extracellular potentials and accounts for the laminar structure and distance-dependent connectivity of our network.

In this simplified model, we ignore heterogeneity in spike-LFP responses *H*_*i*_, of individual presynaptic cells *i* ∈ *X* located within a spatial bin *b*, i.e., *H*_*X*_ ≡ 〈*H*_*i*_〉. *H*_*i*_ corresponds to the extracellular potential resulting of synaptic activation of postsynaptic populations of cells *j* ∈ *Y* from a spike in cell *i* at time τ = 0. We also assume that *H*_*X*_ is invariable across presynaptic bins, and encompasses the overall distance-dependent connection probabilities and connection delays in the network.

The calculation of $H_{X}\left(\vec{\Delta },\tau \right)$ involves a number of steps. We first estimate the spatially averaged extracellular potential $\varphi_{j}\left(\vec{\Delta },\tau \right)$ resulting from a single synapse activation at a time τ^*s*^ of the ball and stick neuron positioned at the center of a reference bin, for excitatory and inhibitory input. Electrode contact point locations **r**_γ_ are defined at the centers of each square spatial bin indexed *b* (see above). With rotational symmetry around the *z*−axis and periodic (torus) connectivity of the network, we compute extracellular potentials at the unique subset of bin center-to-center distances $r\subset \left\{\left|\vec{\Delta }\right|\right\}$ up to the maximum distance $\sqrt{2L^{2}}$, where *L* denotes the side length of the network layers, and $\left\{\left|\vec{\Delta }\right|\right\}$ the complete set of center-to-center displacement vector lengths from reference bin to all spatial bins. We utilize built-in functionality in LFPy to perform spatial averaging (cf. Equation 6 in Lindén et al., 2014), assuming square contact points parallel to the horizontal *xy*−plane with side lengths equal to the bin width Δ*l*^ϕ^. In a following step we compute the average out-degree (number of outgoing connections of neuron *i*) $K_{X}=\underset{Y}{\sum }N_{Y}c$ for *X* ∈ {EX, IN}, where *c* denotes the overall connection probability between *X* and *Y* (cf. Table 2 which also gives *K*_STIM_ as a fixed parameter). With the distance-dependent connectivity ϵ_*YX*_(*r*) used for each presynaptic population and out-degree *K*_*X*_ we compute the number of activated synapses (denoted by *K*_*r*_) in each spatial bin at a distance *r* from the reference bin (including *r* = 0) by evaluating *p*_*YX*_(*r*) at the bin center points. The average connection delays from the reference bin to other bins are approximated as $d_{YX}\left(r\right)=d_{YX}^{0}+r/v_{YX}$, where $d_{YX}^{0}$ denotes a constant delay offset and *v*_*YX*_ the conduction speed of action potentials in the network, with values given in Table 2. With the elements of these steps in place (single-synapse LFP responses across bins, bin-wise number of activated synapses and delays), we construct $H_{X}\left(\vec{\delta },\tau \right)$ as function of *r* as:

(7) $$\begin{array}{l} H_{X}\left(\vec{\Delta },\tau \right)=\underset{r\in \left\{\left|\vec{\Delta }\right|\right\}}{\sum }K_{r}\cdot \left(\delta \left(d_{YX}\left(r\right)\right)*\varphi_{j}\right)\left(\vec{\Delta },\tau \right)\;, \end{array}$$

where δ(·) denotes the Dirac delta function. Note that we sum over all elements *r* in $\left\{\left|\vec{\Delta }\right|\right\}$.

#### 3.5.8. LFP Output

Each *H*_*X*_ is calculated at a spatial resolution Δ*l*^ϕ^ and temporal resolution of *dt* (as in the network, cf. Table 2) for a total duration of 2τ^*s*^, with synapse activation time at time τ^*s*^. An identical spatial and temporal binning resolution is also used for spike events entering in Equation 1. The spike rates in each bin are filtered by a length Δ*t* normalized boxcar filter using the scipy.signal.lfilter method prior to the convolution with the corresponding LFP kernel. Otherwise a temporal shift between the spatiotemporally binned spiking data (cf. Section 3.1) and the downsampled LFP in the visualization occurs. Discrete convolutions are incorporated using numpy.convolve and scipy.signal.convolve2d methods in Python. The final LFP signals are low-pass filtered and downsampled to the time resolution Δ*t* of our preprocessed network output as described in Hagen et al. (2016) in order to simultaneously show both datasets in VIOLA. Output is stored in a pure-text format as described in Section 3.1.

Table 3 summarizes the parameter values for the LFP predictions.

### 3.6. Software Summary

All source codes of the tool VIOLA, the example network model and the processing of model output are hosted at https://github.com/HBPVIS/VIOLA (SHA:a39cb64). We simulated the example network (topo_brunel_alpha_nest.py) with NEST v2.12.0 and Python v2.7.11. Further processing and plotting of Figures 1 and 2 (nest_preprocessing.py) also relied on Python with numpy v1.15.1, SciPy v0.17.0, and matplotlib v2.2.3. LFP signals (fake_LFP_signal.py) were computed using NEURON v7.5 and LFPy v2.0.0 (http://lfpy.github.io). We visualized the neuronal activity with VIOLA using the Google Chrome browser, version 58.0.3029.110 (64-bit). VIOLA used JavaScript V8 5.8.283.38 with the 3D library three.js of revision 87, including WebGL and HTML5 build in the browser and Node.js v4.8.3. For colors, VIOLA used Chroma.js in the version 1.3.4.

Screenshots from VIOLA for the other figures were taken with Kazam-“NCC-80102” v1.4.5, and combined in Microsoft PowerPoint 2013.

## 4. Discussion and Outlook

The present study introduces four 2D and 3D visualization concepts, or views, for the interactive visual analysis of the activity of spiking neuronal network simulations, and a reference implementation for these views named VIOLA (VIsualization Of Layer Activity). VIOLA is an interactive web-technology based visualization tool designed to fit in between simulations and subsequent in-depth data analysis, and exemplifies key concepts of the information-seeking mantra by Shneiderman (1996) and the paradigm of coordinated multiple views (Wang Baldonado et al., 2000). The main application areas are the rapid validation of simulation results and the exploration of spatiotemporally resolved data prior to further quantitative analyses. As a use case, we demonstrate the usefulness of the tool with output from a simulation of a layered spiking point-neuron network model that incorporates distance-dependent connectivity. The use case shows that we can examine a perturbation of ongoing network activity caused by a temporally and spatially confined stimulus. The duration and the spatial spread of the event are quickly assessed with the help of multiple simultaneously displayed views.

In contrast to other visualization tools for simulated network output, for example VisNEST (Nowke et al., 2013, 2015), SNN3DViewer (Kasiński et al., 2009), ViSimpl (Galindo et al., 2016), and Geppetto or more generic multi-view tools like GLUE, the interactive JavaScript- and WebGL-based visualization integrates data analysis methods in a web application, thereby achieving mobility and deployability. Our approach builds on visualization concepts known from the literature for data of similar structure (reviewed in the section), but advances the concepts and adds interactivity and animation. For example, views 1 and 2 compare to series of snap shots (as in Mehring et al. 2003; Yger et al. 2011; Voges and Perrinet 2012; Keane and Gong 2015), but are here enhanced by the possibilities to show raw or preprocessed data, to specify visualization parameters interactively, and to provide a 3D and temporally animated view on the multi-dimensional data. View 4 presents a new concept combining 2D spatial and temporal resolution of multiple neuron populations, all shown simultaneously. This data representation delivers a wealth of information, but, to circumvent occlusion and instead expose interesting features of the data, it relies on interactive usage. The code of the reference implementation is open source and available in a public repository (https://github.com/HBPVIS/VIOLA) together with the revision history and documentation. The present work uses the simulation code NEST to generate the data but the VIOLA implementation is completely independent of the former. The JavaScript code defines a standalone application (accessible at http://hbpvis.github.io/VIOLA) and interpretable by the browser running on the client device. In the last decade, JavaScript-based visualization got more and more versatile especially fostered by the introduction of HTML5 and its canvas environment. Furthermore, the development of WebGL enables the access to GPU-accelerated 3D rendering in the browser. Beside limitations regarding memory and access to low-level program control (as needed for controlled use of multi-threading), JavaScript offers the opportunity for simple deployment and handling of external libraries and dependencies. Unfortunately, JavaScript-based implementations may fail on certain browsers as browsers still differ in their interpretation of JavaScript and in the degree of following the HTML standard. Nevertheless, there are free browsers available that support these technologies for most operating systems. Therefore, this work explores the decision to use web-based technology to offer an easy-to-deploy tool for the visualization of dynamic simulation data. As a consequence, the widely used combination of high-level functions for data analysis and visualization available in for example the Scientific Python ecosystem (SciPy^14^) can no longer be used. Software development and deployment are, however, integrated with minimal effort and no computational resources are required on the server: researchers immediately profit from progress on the development platform. Furthermore, due to the web-technology and the minimal requirements on the client, web portals can embed the application as a visualization back end; a prerequisite for the idea to create centralized ICT (Information and Communication Technology) infrastructure for neuroscience. One such portal is currently being developed by the European Human Brain Project, named the HBP Collaboratory^15^. Another ongoing effort is the Neuroscience Gateway^16^ (Sivagnanam et al., 2013). Online embedding opens the possibility to accompany interactive visualization with server-side preprocessing steps and a database integration, in particular for simulation output being generated on the portal itself. This advances the goal of the HBP Collaboratory to provide a fully digitized workflow from data representation over model construction and simulation to model validation (Senk et al., 2017). We argue that interactive visual analysis of simulated data is an obvious feature of a collaboratory, in addition to non-interactive script-based plotting relying for example on matplotlib.

The reference implementation enables loading of data files via explicit user requests, either using a file browser or drag-dropping the files in the web browser. If data processing and storage were handled on the server-side, SQL-like database queries could restrict communication to only the data needed for the different view instances. Communication does not have to be limited to the raw data. Binning operations similar to those performed in our preprocessing steps can be handled by the database in a straightforward manner, and could also be performed in parallel. The data format HDF^17^ would also be an option to store and access large amounts of raw and preprocessed data with improved performance in terms of speed and compactness compared to the currently used text format.

Inherent in interactive visualization is the problem of reproducibility. The raw data are insufficient to reproduce the visuals, only in combination with the full collection of GUI parameters adjusted by the researchers is the data set complete. In the same way as experimental and simulated data need to be enriched with metadata in order to uniquely specify their origin and enable reuse (Zehl et al., 2016), the visuals need to be enriched with the parameters of their creation. This new type of metadata could be stored in a database.

The JavaScript implementation imposes other shortcomings. One major shortcoming is its limited capability for numerical analysis. While the math.js^18^ library provides a number of basic math functions and support for symbolic operations, complex numbers and arrays (matrices), the JavaScript libraries are not comparable to SciPy which provides an ecosystem of fundamental tools and methods encountered in mathematics, engineering, and science. VIOLA implements the function computing the spatial correlation of neuronal activity from scratch (not shown). This approach has two conceptual weaknesses. First, the speed and accuracy of such functions are hampered by the fact that there is little native support for advanced mathematical operations, like the Fast Fourier Transform (FFT). Second, there is no separation between the code carrying out the statistical analysis and the code performing the visualization. This cuts visualization off from the rich set of analysis tools developed by the community and their reliable implementations, for example as collected in the Elephant package^19^. Future work needs to disentangle numerics from visualization code as separate building blocks in a visual analysis workflow.

As VIOLA's main focus lies on responsive interactive visualization, the reference implementation uses WebGL for all views. Prior tests exposed the low efficiency of the Document Object Model (DOM) as used in Scalable Vector Graphics (SVG) based visualization libraries such as d3.js as well as its high memory consumption. This led to the decision to the sole use of WebGL rendering, which has the limitation that external tools are required for generating screen shots and screen casts; vector graphics can neither be recorded nor exported. For the 2D views, an additional implementation based on the HTML5 support of SVG graphics can be added and used for the export of vector-based image material. For extracting vector-based material from the 3D views, WebGL and its access to the underlying rendering pipeline can be facilitated. The 3D scene can be exported to be viewed in other 3D programs. To this end, three.js (as used in the reference implementation) offers export functionality for Wavefront OBJ file format, one standard for 3D content. The alternative is to extract the rendered scene prior to rasterization and use these data to generate a SVG or postscript-based representation similar to the operation of the C library gl2ps^20^. Nevertheless, any export mechanism needs to facilitate means of reproducibility. In particular metadata such as simulation and visualization parameters, time stamps, viewpoint angle and position etc. need to be bundled with the raw visualizations. As direct file writes may not be possible in a client side JavaScript application, one solution is server-based rendering and storage based on visualization parameters being communicated from the client back to the server. The resulting server-side rendered images are then stored as provenance information. Another option for reproducible visualization outcome is to only store the previously mentioned visualization parameters in the database, such that the client-side visualization application can be set back into the original captured state. If these parameters are captured over a longer period, the resulting data can ease the regeneration of content for demonstration purposes or a *post hoc* video rendering.

While we here develop our arguments along model data, the different views and the reference implementation are equally suited for the exploration of experimental data. Our model network describes a neuronal layer covering a 4 × 4 mm^2^ patch of cortical tissue. Electrophysiological measurements of neuronal activity with the Utah multi-electrode array from Blackrock Microsystems sample both spiking activity of individual cells and population LFPs across near 4 × 4 mm^2^ of cortex (Milekovic et al., 2015; Torre et al., 2016b; Denker et al., 2018). Denker et al. (2018), for example, classify complex spatiotemporal patterns in the LFP beta phase of Utah-array recordings in monkey motor cortex during a delayed reach-to-grasp task. Their Figure 3B shows sequences of snapshots of the pattern evolution in time similar to the time frames shown here for spiking activity (Figure 7). Townsend et al. (2015) also demonstrate snapshots of complex patterns measured in monkey visual cortex in their Figure 2, and in Figure 4B they display the LFP phase together with the spiking activity. Such experimentally obtained discrete spike events and analog LFP signals can be simultaneously visualized with VIOLA, as shown by views 2 and 4 (Figures 3 and 4) for model data. The visualization of the aforementioned experimental data would not require any changes to the reference implementation. As VIOLA lends itself to assess spatial and temporal relationships between spikes and LFPs, the data considered in a study by Nauhaus et al. (2009) might also be an interesting example to visualize. The authors show spreading depolarizations in the LFP amplitude triggered by spikes in monkey visual cortex (see their Figure 2). Such events could be visually analyzed to evaluate the spatial spread and the duration of the perturbation, similarly to the VIOLA use case described in Section 2.2 to visually assess the effect of an external stimulus on the network activity. Apart from *in vivo* recordings with the Utah array, data obtained with other multi-electrode arrays used for *in vitro* experimentation on neural tissue or cell cultures (Massobrio et al., 2015) can also be visualized with VIOLA.

Measurement modalities other than spikes and LFPs are of interest as well. One common experimental method is Ca^2+^ imaging which may infer changes in intracellular [Ca^2+^] of neurons in superficial (Grienberger and Konnerth, 2012) and deep layers (Ouzounov et al., 2017), while another method is voltage-sensitive dye imaging (VSDi) that measures membrane-voltage time-derivatives in surface-proximal tissues (Chemla and Chavane, 2010). With modifications to existing views or new view implementations, VIOLA can also represent this type of spatiotemporally resolved data. In particular the 3D visualization types incorporated in the present views 2 and 4 are well-suited to represent the changes in intracellular Ca^2+^ ion concentrations across different cell bodies from 2- and 3-photon volumetric Ca^2+^ imaging in neural tissue. Within view 2 the visual representations of each cell's concentration can be set to a depth and position in the horizontal plane according to its image stack position in the raw imaging data. Units with baseline Ca^2+^ concentrations may then be hidden, and increasing levels can be visualized by scaling the box sizes as we have demonstrated with spike-rate data. A view similar to view 4 could show time-varying ion-concentrations of individual units as 3D tube plots where the tube diameter at a given time is proportional to a unit's Ca^2+^ concentration. As VSDi imaging data (typically) lacks depth-information, color-image plotting can be applied similar to what we utilize here to show LFPs in our 3D view implementations. In addition, the multi-view aspect of visualization enables the combination of spatial representations with more abstract non-spatial representations of neuronal activity, as reviewed in Section 1. Experimental data or model data from a different origin than described in this work require a conversion from the original data format to the data format readable by VIOLA (see Section 3.1). A freely available experimental data set that can be inspected visually with VIOLA are, for example, the published Utah-array recordings described by Brochier et al. (2018).

The concepts developed here advance the visual exploration of data from cortical networks at cellular resolution. If the reference implementation finds more widespread interest it can be further developed by a community driven approach as all requirements like a proper licensing and a suitable development platform are in place, the primary purpose, however, is to serve as a living supplement to this publication. Creating a common web portal for the collaboration of neuroscientists is a central long-term goal of the Human Brain Project. In this endeavor our study contributes knowledge on how a user interface for visual exploration needs to be designed and on the proper layout of the software stack at the troubled transition point between data processing and visualization.

## Author contributions

JS, EH, CC, TK, MD, and BW conceived and designed the study. JS, EH, CC, and BW designed the various types of visualizations. CC implemented the first version of VIOLA and JS co-developed the tool. JS and EH implemented simulation, preprocessing, and analysis code for the example network. EH incorporated LFP predictions. JS ran all simulations and created the figures. JS wrote the first version of the paper. EH, BW, MD, CC, and TK co-wrote the paper.

## Conflict of interest statement

The authors declare that the research was conducted in the absence of any commercial or financial relationships that could be construed as a potential conflict of interest.

## Footnotes

^1^http://blackrockmicro.com

^2^https://moose.ncbs.res.in

^3^http://nest-simulator.org

^4^https://www.openinventor.com

^5^http://www.geppetto.org

^6^http://glueviz.org

^7^https://web.archive.org/web/20180305220332/http://www.opendx.org

^8^http://www.json.org

^9^https://www.vis4.net/blog/posts/mastering-multi-hued-color-scales

^10^https://threejs.org

^11^https://nodejs.org

^12^http://www.python.org

^13^http://lfpy.github.io

^14^https://www.scipy.org

^15^https://collab.humanbrainproject.eu

^16^http://www.nsgportal.org

^17^https://www.hdfgroup.org

^18^http://mathjs.org

^19^http://elephant.readthedocs.io

^20^http://www.geuz.org/gl2ps

## Supplementary material

Videos 1–5 We provide one movie demonstrating the interactive usage and multi-view aspect of the tool VIOLA (Video 1) and one movie for each of the four views of VIOLA (Videos 2–5).
